# Interactional Fingerprints Offer Accessible, Rapid, and Qualitative Characterization of Graphene Oxide

**DOI:** 10.1021/jacs.5c05355

**Published:** 2025-07-09

**Authors:** Blanca Ivonne Vergara-Arenas, Esmé Shepherd, Ivan Alfaro, Edward Cross, Huong Le, Andrew J. Surman

**Affiliations:** Department of Chemistry, 4616 King’s College London, London SE1 1DB, UK

## Abstract

Graphene-based materials (GBMs), including graphene oxide and graphene, are atomically thin materials with great promise, but efforts to realize their potential have been hampered by inconsistent material supply and the lack of rapid, accessible characterization methods. Here we present a new approach to characterization, based on surface interactions with a series of probe molecules. We demonstrate that our method facilitates qualitative comparisons of graphene oxide materials—for example, if batches of material differ—an accessible quality control method for materials producers and users alike, using widely available instruments. Furthermore, we show that in some circumstances, we may quantify systematic differences, such as surface modifications. Since many applications of materials depend on surface interactions, we suggest that this approach is applicable beyond quality control as a more direct approach to probe material surfaces and a valuable alternative to instrumental methods in GBMs and beyond.

## Graphical abstract

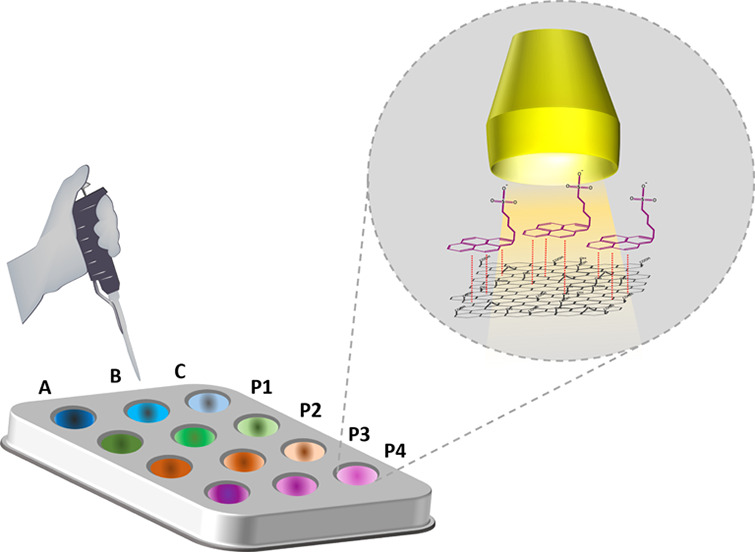

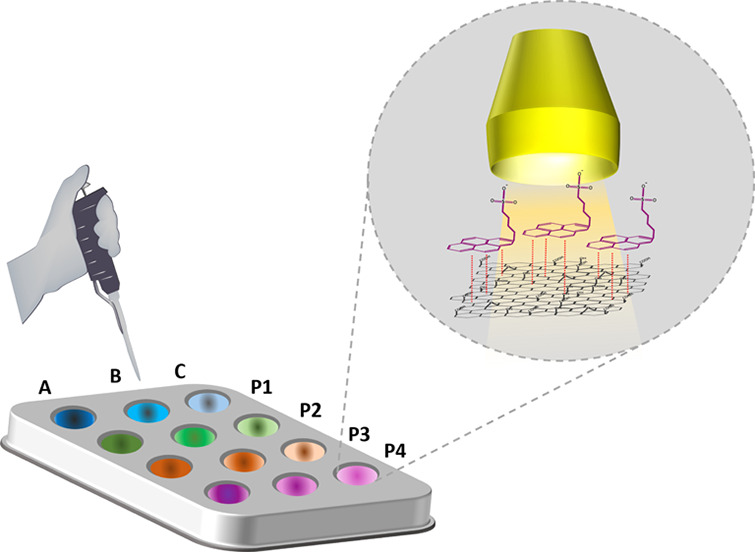

## Introduction

Graphene, graphene oxide, and other “graphene-based materials” (GBMs) are materials based on a single layer of carbon, each only one atom thick.^1,2^ Following the isolation of graphene in the early 21st century,^3,4^ they have been hailed as “wonder materials”, and vast sums have been invested in developing applications.^5^ This promise is real: atomically thin, and “few-layer”, flakes of material have very distinct and exciting electronic and mechanical properties (c.f. “bulk” materials), with potential applications in electronics, clean energy, and sensor technologies.^6^ Over 15 years later, though, much of this promise has not yet been realized.^5^ Anecdotally, it has been known for some time that a barrier to the adoption of GBMs has been an unreliable supply of materials, leading to irreproducible results: users complain that using the “same” material, purchased from the same supplier, can produce distinct results. A recent study surveying commercially available graphene materials brought the extent of the problem to light: the majority of samples analyzed contained less than 10% single-layer graphene, with the authors summing up that *“producers are labeling black powders as graphene and selling for top dollar, while in reality they contain mostly cheap graphite”*.^7^ A 2023 study of graphene oxide similarly found only a small fraction *“deliver approximately what they display on the label or brochure”*.^8^ The authors of those studies, along with widespread comment,^9,10^ suggest that this issue represents a barrier to realizing GBMs’ potential and suggest quality control and characterization by both producers and users of the material as the remedy.

Until recently, there had been little agreement on what constitutes appropriate characterization. This was partially addressed by the publication of an International Standard for graphene characterization in 2021.^11,12^ This approach involved rigorous characterization using a range of instrumental procedures, including Raman spectroscopy, electron microscopy (either Scanning Electron Microscopy, SEM, or Transmission Electron Microscopy, TEM), Atomic Force Microscopy (AFM), and measurement of surface area using gas physisorption (via the Brunauer–Emmett–Teller method, BET). While no corresponding International Standard has yet been published for graphene oxide, which has more complex surface chemistry, an analogous “gold standard” approach would include the same with the addition of X-ray Photoelectron Spectroscopy to determine the abundance of carboxylic acid, alcohol, and epoxide groups (as demonstrated in a recent survey of supply).^8^ Such characterization is desirable but is typically slow, costly, and requires specialized personnel, making it inaccessible to many laboratories (both academic and industrial).

More accessible characterization methods are clearly needed for materials producers and users alike to perform quality control (QC) testing. Such methods should be rapid (hours, not days or weeks), cheap (few dollars, not thousands of dollars), use only widely available apparatus, and be feasibly performed by technical staff in a nonspecialist lab setting or factory.^12^ Importantly, commentators note that routine methods need not provide the same level of comprehensive structural and chemical insight as *“gold standard”* methods to be practically useful: qualitative comparison of materials and identifying changes is sufficient for many QC needs—that is, to allow the user to ask, *“Is this material like other batches?”* ^12^ Some materials users opt to apply a subset of gold standard techniques and/or methods, such as Dynamic Light Scattering (DLS),^13^ p*K*_a_ measurement,^14^ thermogravimetric analysis (TGA),^15^ infrared spectroscopy,^16^ NMR relaxation,^17^ and covalent attachment of dyes to quantify surface functional groups,^18^ but most of these have not served to fulfill material users’ needs for a rapid, cheap, accessible method covering a range of properties, and no particular single approach has been widely adopted.

This work focuses on the rapid characterization of GO, as it is a particularly challenging GBM produced by a range of manufacturing methods that yield wide variations in its surface chemistry,^8,19^ with many applications depending on the effect this has on surfaces’ molecular recognition and aggregation properties.^20,21^

Here we demonstrate a new approach to understanding graphene oxide (GO) materials based on the interaction of an array of probe molecules with GO surfaces, as outlined in Figure 1. While they have not previously been applied to material surfaces, our approach is similar to other supramolecular sensor/probe arrays with optical detection:^22–24^ when molecular probes interact with aqueous GBM dispersions, their signal (fluorescence) is quenched, providing a ready and rapid means to measure interaction. One probe molecule’s interaction cannot characterize the many variables describing a material. However, if a series (or “array”) of different probes interact with distinct sites, with distinct affinities, the distribution of responses constitutes an “interactional fingerprint” (see Figures 1 and 3A) incorporating information on many variables. Deconvolution of fingerprints by multivariate analysis methods provides a readout of sample composition: for our purposes, producing a 2D map where adjacent materials are similar. A small range of GBM characterization methods based on noncovalent interactions has already been reported. These include adsorption for surface area measurement (using gases,^25^ methylene blue dye,^26^ and/or dopamine,^27^ though questions remain over quantification using soluble probes)^27^ and fluorescence quenching for optical microscopy.^28^ These methods are limited, as they only provide information on one variable (size), and this may apply only in the unlikely event that other variables do not change; so far, few studies report their routine use. A paper on the electrochemical study of phenols’ surface interactions at GO surfaces suggests this may be used to understand GO composition,^29^ but we find no citing work following this suggestion. This work provides an alternative approach that can be applied to GO QC and to material surfaces more generally.

**Figure 1:**
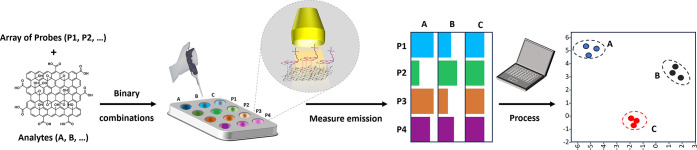
Concept and workflow for a rapid interactional fingerprinting assay. Each of a series of probe molecules is mixed with a GO dispersion sample (analyte) in binary combinations within each well of an appropriate microplate. Where probes interact with the GO surface, a change in the probes’ fluorescence can be observed (quenching); a series of these responses can be considered an *“interactional fingerprint”*. Deconvoluting this complex fingerprint data for a series of GO samples (multivariate analysis, e.g., by Principal Component Analysis, PCA, or Linear Discriminant Analysis, LDA) can provide a readout or “map”, in which similar materials are adjacent.

## Results

### Establishing a Probe Array

To construct a probe/sensor array, we require probe molecules that (i) interact with the analyte, GO surfaces, and (ii) provide a measurable signal change when they interact. Fluorescent probes are well-suited, as interaction with GO typically quenches fluorescence via an energy transfer mechanism,^30^ and the means to measure this change (fluorescence microplate readers) are widely available. An ideal probe to interact strongly with a GO surface (Figure 2A) in aqueous dispersion would include (Figure 2B) a flat aromatic moiety for hydrophobic interaction with graphene surfaces and a hydrophilic group to ensure solubility (and modulate interaction with polar groups). Such a design mirrors that of some reported graphene “dispersants”^31^ (surfactants to mediate aqueous dispersion of insoluble graphene flakes), and specifically the molecule is illustrated in Figure 2B (**P1**).^16^ Although fluorescence is not relevant to dispersants (and not typically reported), we show here that GO interaction quenches these molecules’ emissions (Figure 2C). A number of species already known to interact with GO surfaces are also fluorescent, offering similar promise as probes.^28^

**Figure 2:**
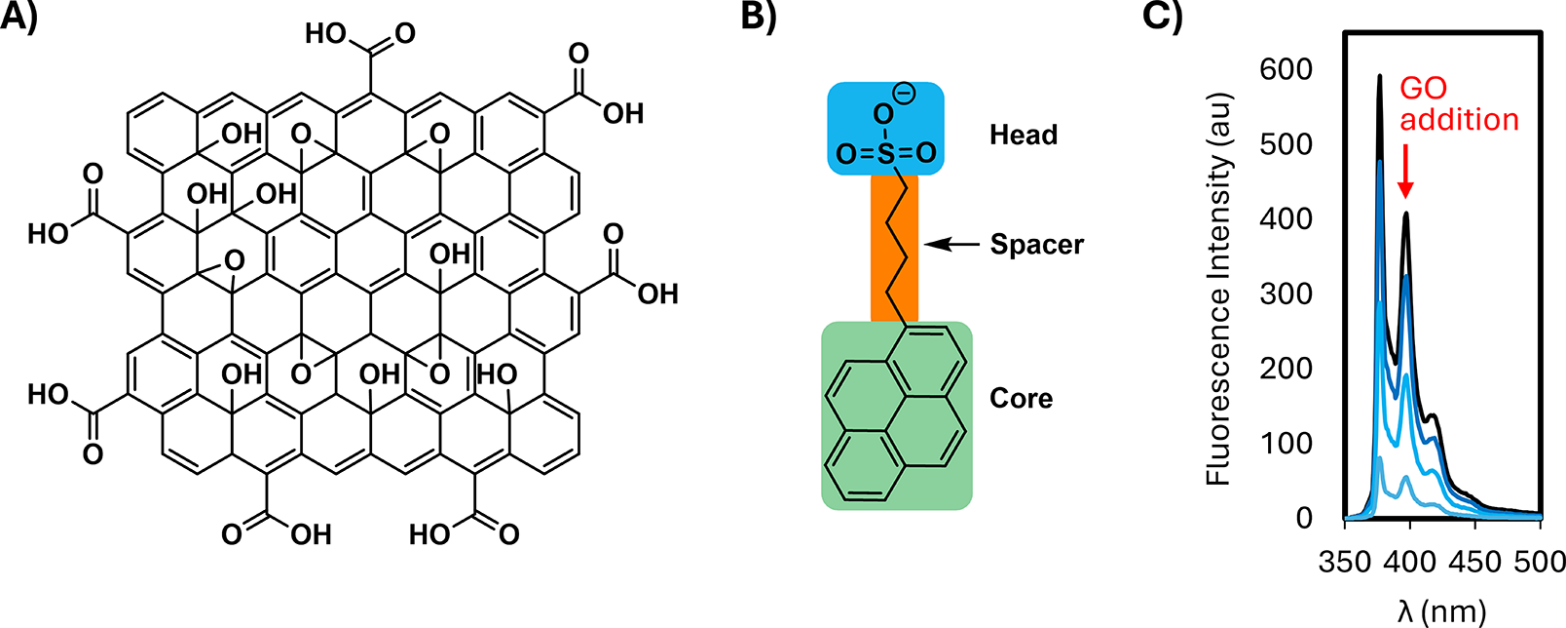
Establishing probe quenching through interaction with GO. A suitable probe for a GO surface (A) should incorporate the moieties shown in (B), and its fluorescence must be quenched on interaction with GO surfaces (C, quenching of **P1** on addition of GO).

For this study, we assembled a library of probes **P1–P10** (Scheme 1), comprising a range of known dispersants and other fluorescent probes. We note that when constructing an array of sensors, the goal is diversity of interactions—in strength and nature—and this library includes molecules expected/known to bind strongly (e.g., **P1–P5**, known as dispersants)^16^ and molecules that may bind to different extents (e.g., **P6–P10**), providing diversity. Most are commercially available, and the remainder (**P1**, **P5**) were readily synthesized in a few steps using established procedures (see Supporting Information).^16,32^ All are fluorescent (see Section S2.2), and their fluorescence is quenched on interacting with GO.^30^

**Scheme 1:**
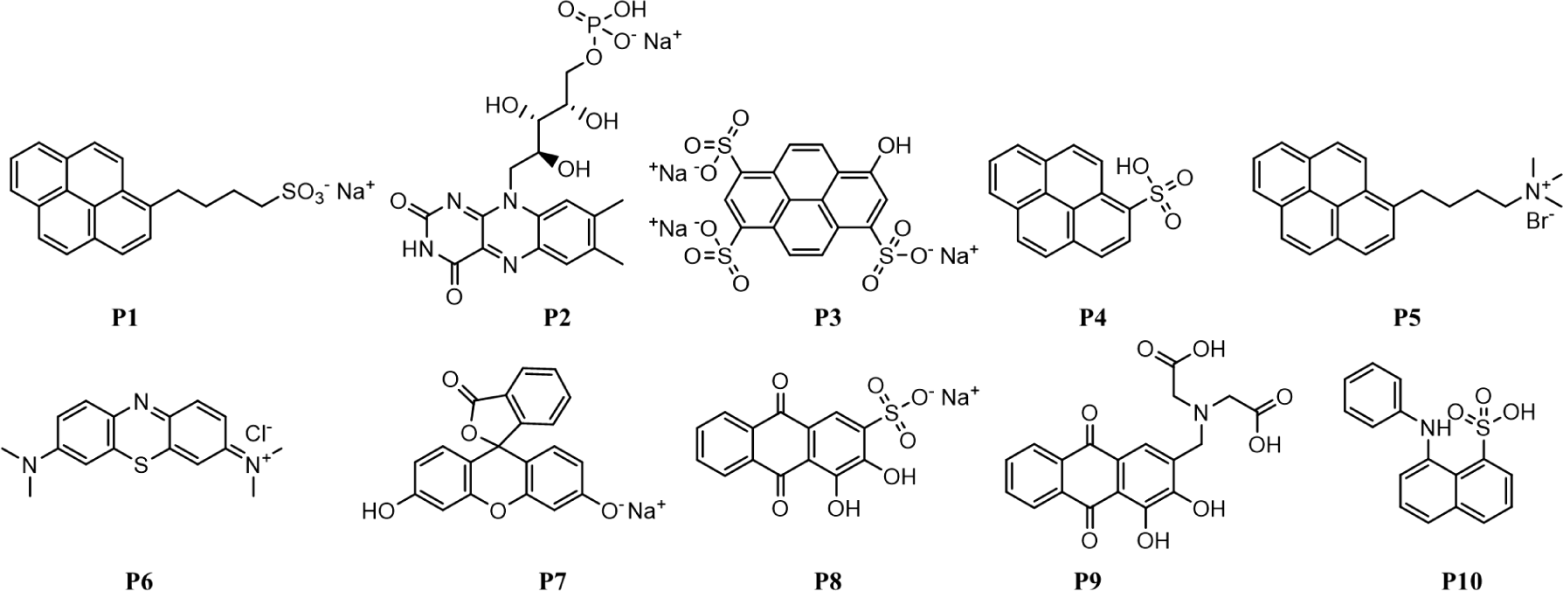
Library of GO-Interacting Probes. Most Probes Are Available Commercially, Except for P1 and P5 (See Supplementary Information for Synthesis)

### Library of GO Commercially Available Materials

To test our assay, we purchased a diverse selection of commercially available GO materials from a variety of suppliers. Along with 8 samples sold as graphene oxide, we also added samples described, respectively, as “*Ammonia Functionalized Graphene Oxide”* (**GO­[B]**) and *“Carboxylic acid enriched Graphene Oxide”* (**GO­[J]**). Most samples were purchased as dispersions, and those that were bought as flakes were dispersed in water.

We performed a range of orthodox characterization on our library of GO materials (XPS, Raman, SEM, IR, UV, ICP_MS), as summarized in Table 1 (see Section S4 for full results). Unlike graphene, which has a single structure varying only in dimensions and defects, “graphene oxide” represents a diverse range of materials that can vary in a variety of parameters while still being considered graphene oxide—in particular in the nature and patterning of surface groups (e.g., acid, alcohol, epoxide). The range obtained here is no exception. From XPS elemental analysis, we see that O/C ratios generally fall in the 0.3–0.6 range typical of commercial GO;^8^ some outliers (e.g., **GO­[M]**, **GO­[D]**) have very low oxygen content (consistent with “reduced GO” or graphene/graphite). As expected, significant amounts of nitrogen were observed in the XPS survey of the ammonia-functionalized GO (**GO­[B]**; 2.6% N), with negligible amounts observed in most other samples (<0.6, which may reflect traces of buffers). By SEM we observe a range of flake sizes, while some materials do not appear homogeneous or do not appear to be composed of 2D flakes (see Section S4.5 for images). By Raman spectroscopy we note that some Raman *I*_D_/*I*_G_ ratios vary considerably with most around 1.0–1.4 (consistent with larger surveys)^8^ and some notably lower (e.g., **GO­[D]**, **GO­[L]**, **GO­[M]**); some samples also stand out as lacking observable *I*_2D_ bands (e.g., **GO­[C]**, **GO­[N]**). IR spectra of some samples lack features consistent with GO structure (e.g., **GO­[D]**, **GO­[L]**, **GO­[M]**). ICP-MS studies reveal varying (low) concentrations of metal contaminants; however, as nonalkali metals are below 0.1 mol % of probe concentration in our assays (see later), no impact from these is expected. Importantly, we note that many of these properties vary orthogonally, with no simple relationship apparent, meaning rapid measurement with a single established method is unlikely to differentiate materials clearly.

**Table 1:** Collection of Commercially Available GO Materials^a^

Sample	Sold as	Flake Size (μm^2^)^b^	O/C^c^	*I*_D_/*I*_G_^d^	*I*_2D_/*I*_G_	IR “flat”^e^	“Target GO” range?
**GO[A]**	GO	4.93 (±0.43)	0.44	1.27	0.22	n	Yes
**GO[B]**	Ammonia-functionalized GO	2.31 (±0.28)	0.31	1.37	0.20	n	
**GO[C]**	GO	0.86 (±0.25)	0.47	1.19	*negligible*	n	
**GO[D]**	GO	2.58 (±0.26)	0.05	0.30	0.27	y	
**GO[E]**	GO	0.44 (±0.12)	0.47	1.38	0.17	n	Yes
**GO[J]**	Carboxylic acid enriched GO	0.95 (±0.49)	0.45	1.23	0.29	n	Yes
**GO[K]**	GO	0.89 (±0.24)	0.46	1.40	0.27	n	Yes
**GO[L]**	GO	4.63 (±0.32)	0.32	0.26	0.13	y	
**GO[M]**	GO	4.70 (±0.29)	0.12	0.06	0.39	y	
**GO[N]**	GO	4.73 (±0.29)	0.58	1.36	*negligible*	n	

a Full characterization data and acquisition details are available in Supporting Information.

b Flake size distribution taken from SEM images.

c Elemental ratio of oxygen to carbon (O/C) calculated from XPS survey spectra.

d Intensity ratio of “D” and “G” bands in (*I*_D_/*I*_G_) in Raman spectra.

e Is IR spectrum featureless, inconsistent with material being graphene oxide? (“y” = flat, inconsistent with GO structure; “n” = IR spectrum features consistent with GO structure).

While many of the measurements noted here as outliers are not inconsistent with the broad range of possible GO structures (e.g., lacking observable *I*_2D_ bands in Raman analysis is consistent with higher levels of oxidation), a group of materials is notably similar and falls within the ranges more typically observed^8^ (**GO­[A]**, **GO­[E]**, **GO­[J]**, **GO­[K]**): we will refer to these as “Target GO” throughout the remainder of this paper. This is an arbitrary label, not to be taken as a judgment of “quality”, but denoting the range of similar materials for which we intend to optimize our assay.

### Applying the Probe Array to Map GO Materials’ Interactional Fingerprints

To establish reasonable conditions for applying our array, we performed small-scale tests to determine conditions in which all probes could be observed interacting to some extent with a small number of “Target GO” sample dispersions without completely quenching fluorescence, as well as altering probe concentration, GO concentration, pH, ionic strength, and GO handling (e.g., ″settle times”, following mild agitation, during which a dispersion is homogeneous and the response is reproducible).

Under these assay conditions, we then surveyed the response of our full range of probes to our GO library, with responses shown in Figure 3A: the GO materials’ “interactional fingerprints”. Most of the GO samples produced distinct responses (degrees of probe quenching). The fact that the responses vary orthogonally reflects a range of properties. For example, the response of **P2** to **GO­[K]** and **GO­[L]** is similar, whereas the response of **P6** to the same materials is markedly distinct. It is difficult to interpret this multivariate data set directly. Applying Principal Component Analysis (PCA) to deconvolute the “fingerprint” data, we can map our materials in a 3D plot (Figure 3B; see Figure S1 for a simpler 2D representation). PCA is an unsupervised method (it uses no data on sample identity), yet in the PCA plot, we see that dissimilar materials are distant and clearly resolved, and similar materials (as determined by conventional analysis) are grouped together. Materials differ in all three principal components independently (e.g., **GO­[C]** and **GO­[D]** are close in *PC1* and *PC2* but more distant in *PC3*, reflecting their very distinct surface chemistry), again demonstrating that this mapping is not a response to a single attribute but a qualitative “summary” of the materials’ properties.

**Figure 3:**
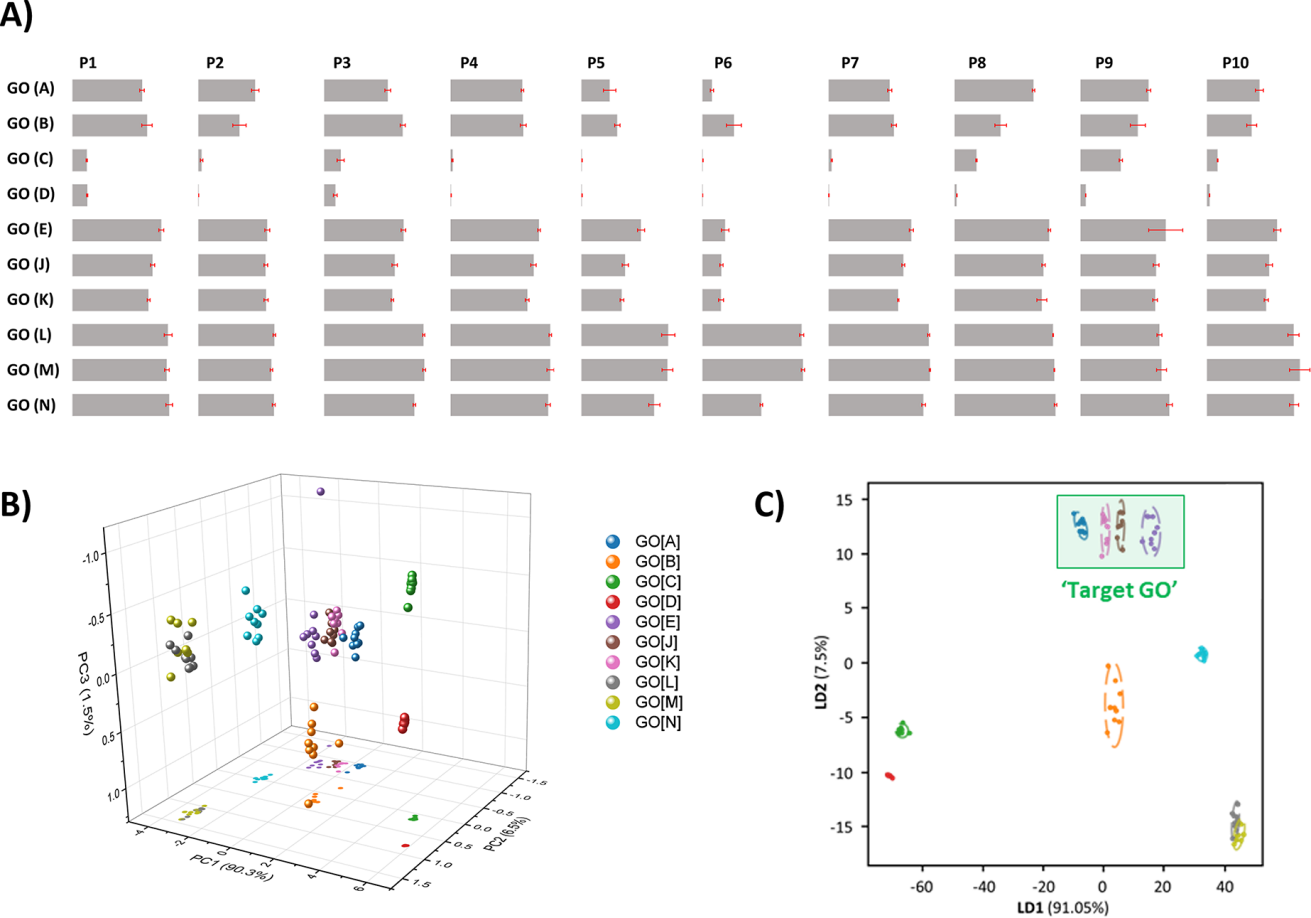
Multivariate analysis of GO library “Interactional Fingerprint” data provides qualitative information on GO materials. (A) Normalized fluorescent response from each probe to each GO sample [see Table S1for excitation/emission wavelengths; average readings from 9 measurements; error bars represent 1 standard deviation; see Table S2for full data]. (B) 3D PCA analysis plot of interaction data from all probes (**P1**–**P10**) with GO library, representing ca. 98.3% of data variance. [Note: PCA is an unsupervised technique. Colors were added for display, and the identity of samples is not included in processing.] (C) LDA analysis of interaction data from all probes (**P1**–**P10**) with GO library. The area in which “Target GO” samples fall is marked: a small subset of LDA space, distant from other samples observed to be more distinct by orthodox analysis. [In panels **B** and **C**, “points” represent the response of a single reading; ellipses represent a 95% confidence limit for each GO material, calculated after multivariate analysis is completed, representing variation on repeated measurement].

Linear Discriminant Analysis (LDA) is a supervised method that incorporates information about which group each data point belongs to **GO­[A]**, **GO­[B]**, etc. to optimize the resolution of the materials. Applying LDA (Figure 3C) provides greater resolution between the different materials, such that almost all samples are clearly resolved in two dimensions (repeated stratified k-fold cross-validation suggests >97% classification accuracy). Even in a simple 2D plot, all samples characterized as resembling “Target GO” (see Table 1) occupy one area of the plot, which is clearly distinct from the space occupied by other materials.

While it is far more accessible than conventional characterization, obtaining readings of responses to our full library of probes (**P1** to **P10**) still requires considerable labor (ca. 30–40 min liquid handling/pipetting dominates the time taken to perform these analyses). By reducing the range of probes used, we find that similar LDA plots are observed (see Figure S2): even using 3 probes (**P2**, **P6**, **P7**; selected by examining data to determine a minimal probe set capable of discriminating this range of materials) yields a qualitatively similar result, while requiring fewer manipulations.

A practical QC method should allow a GO material user to ask, *“Is this material like other batches?”* when obtaining new material or performing quality control to compare a new batch of material with others.^12^ To simulate this, we split our data (on responses to only three probes: **P2**, **P6**, **P7**) using responses to most GO sample data as “calibrants” to train an LDA model and reserved **GO­[J]**, **GO­[L]**, and **GO­[B]** as “test” samples. This models a quality control scenario, allowing us to ask, *“Is this batch like the last?”*. Training an LDA model with this reduced set produced a plot similar to those of other LDA analyses (filled ellipses, Figure 4). Applying this model to the test set, we see that all three samples are placed in an area of the resulting plot that reflects their properties (data in unfilled ellipses, Figure 4): **GO­[J]** falls between the similar “Target GO” samples **GO­[E]** and **GO­[K]**; the ammonia-functionalized GO sample **GO­[B]** is discriminated from all of these, and the outlying sample **GO­[J]** remains identifiable as clearly distinct. For consumers or producers of “Target GO” materials, a new batch of material not falling into the appropriate range, but fitting into the range of material sold as “graphene oxide”, would be clearly observed.

**Figure 4:**
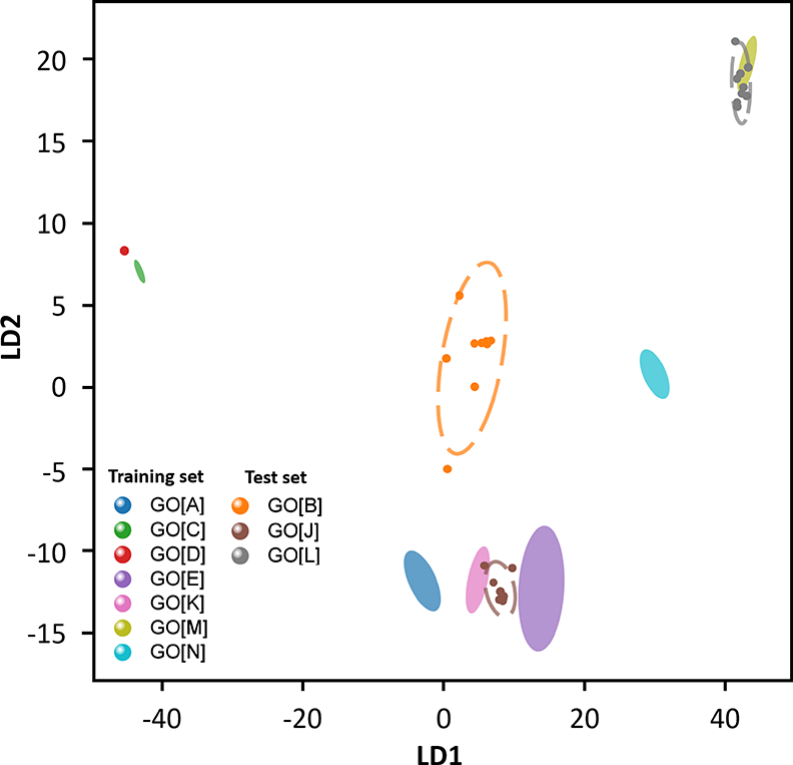
Practical comparison of “new” materials to a “test” or calibration set of GO. LDA analysis of interaction data from a reduced range of probes (P2, P6, P7) with a “test” (or calibration) subset of the GO library (**GO­[A], GO­[C], GO­[D], GO­[E], GO­[K], GO­[M], GO­[N]**), represented as filled ellipses. This LDA model is then applied to a “test” set of GO materials, represented as “points”, which fall in areas adjacent to similar materials. [Ellipses represent a 95% confidence limit for each GO material, calculated after analysis is completed, representing variation on repeated measurement; “points” represent the response of a single reading].

### Applying the Fingerprinting Approach to Extract Quantitative Information

Qualitative analysis is well-suited for many needs. However, in some cases, quantification of a systematically varying property is necessary, particularly when performing modification of a material surface. To test the potential of our method to assess this, we produced a series of modified GO samples from the same starting material, in which differing proportions of surface alcohol groups had been esterified (Section S5) and measured the degree of modification using an established method (see Supporting Information). Performing our assay on the resulting GO materials (**GO­[Mod-A]** to **GO­[Mod-E]**, w/**P1** to **P5**) and subjecting the resulting “interactional fingerprint” data to PCA revealed a progressive shift in *PC1* as the degree of modification increased (see Figure S11). Splitting materials into training (most materials) and test sets, performing PCA on all, and plotting mean *PC1* against the degree of surface modification for our test set, we observed a linear relationship between the degree of modification and *PC1* response (see Figure S11). Using this relationship, we were able to estimate the degree of modification of the test material (**GO­[Mod-D]**, estimated as 39.5% modified; determined as 38% modified). While we note that this series of materials varies systematically only in the degree of modification, this demonstrates the potential for this fingerprinting approach to rapidly assess surface modification. Quantification of other parameters may be possible, but models will likely require training with considerably larger materials libraries.

## Discussion

We have shown that obtaining an “interactional fingerprint” of GO material dispersions, by applying an array of suitable fluorescent probe molecules, can provide useful qualitative information on GO materials. Leading commentators have discussed the need for *“rapid and inexpensive”* QC methods, which *“can be performed on the factory floor”* to *“identify changes in the material”*, and which need not provide all the information yielded by gold standard methods.^12^ We propose that our fingerprinting approach is well-suited to this role, here allowing us to rapidly differentiate GO samples (e.g., the analysis in Figure 4 could be readily performed in around an hour, given practice: ca. 20 min pipetting; 15 min ″settle time”, 2 min data acquisition, 15 min data preparation, and 2 min data processing; c.f., several days to complete the series of experiments required for orthodox characterization). As noted above, this method is not suitable for comprehensive characterization of every parameter that might be measured by (slow, expensive) “gold standard” methods; it is not intended to replace them but to complement them and provide practical alternatives (and may be combined with other partial characterization methods as appropriate). We note that, unlike orthodox approaches,^12^ this can be achieved using only simple apparatus/materials (pipettors, microplate reader) and commercially available materials (probes, reagents, GO standards), making it accessible well beyond specialized laboratories. An assay in which nanomaterials are handled as aqueous dispersions is also preferable for safety reasons.

The fingerprinting method we present here is optimized for one group of materials (arbitrarily labeled as “Target GO”), allowing us to distinguish between these and other GO materials that are available commercially. Given relevant material samples (“standards”), this kind of assay can readily be tuned for a range of challenges (by altering probe choice, probe concentration, GO concentration, pH, ionic strength, etc.). This may be helpful, for example, to determine finer variations between *“in spec”* and *“out of spec”* materials, as might be required by manufacturers for QC, or more broadly to distinguish between different classes of GO/GBM.

Many applications of materials depend on surface properties, such as sequestration of pollutants,^33^ metal extraction,^34^ compounding in hybrid materials,^35^ and photocatalysis. Indeed, GO surfaces are often used as a probe for other analytes or as a platform for sensing.^36^ Our concept is a reversal of this, focusing on understanding the material through its recognition properties. Beyond simple QC, we propose that our approach is well-suited to assess the surface properties of a range of materials, offering insights complementary to those available from more orthodox microscopy- and spectroscopy-based characterization (given appropriate optimization and material standards). Furthermore, the broad and untargeted nature of the “fingerprint” data produced is well-suited to machine learning or artificial intelligence-based interpretation. While the limited volume of data available in this study is insufficient to train machine learning models, this approach offers further potential for insight as the interactional fingerprinting of materials develops.

## Supplementary Material



## Notes

Includes Experimental procedures, Figures S1–S17, and Tables S1–S5. The commercially available graphene oxide samples were obtained in the UK, sourced from companies based in the UK, Europe, and nearby countries, and analyzed without further purification beyond the described protocols (PDF)

## Abbreviations

GBMs: Graphene-based materials
GO: Graphene oxide
QC: Quality control
PCA: Principal component analysis
LDA: Linear discriminant analysis
SEM: Scanning electron microscopy
TEM: Transmission electron microscopy
AFM: Atomic force microscopy
BET: Brunauer–Emmett–Teller method
DLS: Dynamic light scattering
XPS: X-ray photoelectron spectroscopy
