# Supplementary material for: Interactional Fingerprints Offer Accessible, Rapid, and Qualitative Characterization of Graphene Oxide
Source: J Am Chem Soc. 2025 Jul 9;147(29):25471–7. doi: 10.1021/jacs.5c05355 (PMC12291440; doi:10.1021/jacs.5c05355)
Supplement: Supplementary file 1 [file ja5c05355_si_001.pdf]

Supplementary Information

Interactional fingerprints offer accessible, rapid, qualitative characterisation of graphene oxide.

B. Ivonne Vergara-Arenas,[1] Esmé Shepherd,[1] Ivan Alfaro,[1] Edward Cross,[1] Huong Le,[1] and Andrew J. Surman.*[1]

[1]Department of Chemistry, King's College London, SE1 1DB, UK

*Correspondence: andrew.surman@kcl.ac.uk.

Contents:

1. General Materials and Methods. 3
2. Synthesis and Characterization of Probes. 4
   2.1 Synthesis of **P1** and **P5.** 4
   2.2 UV-vis and fluorescence spectra of probes. 6
   2.3 Interactional fingerprinting assay. 10
3. Multivariate analysis of fingerprint data. 12
4. Orthodox characterisation on our library of GO materials. 14
   4.1 UV-vis. 14
   4.2 FT-IR. 15
   4.3 XPS. 16
   4.4 Raman spectroscopy. 20
   4.5 SEM. 21
   4.6 ICP-MS 24
5. Modification of graphene oxide surface, by alkylation of surface alcohol groups. 25
6. Quantification of degree of surface modification using established quantitative methods. 26
7. Quantification of degree of surface modification using a modified 'interaction fingerprint' assay. 28
8. Other orthodox characterisation of graphene oxide modified with acetyl group. 29
   8.1 UV-vis. 29
   8.2 FT-IR. 30
   8.3 XPS. 31
   8.4 Raman spectroscopy. 34

1. General Materials and Methods

1-pyrenebutanol, 1-pyrenesulfonic acid **P4**, Phosphate Dibasic Heptahydrate and Potassium Phosphate Monobasic Monohydratewere were obtained commercially from Sigma-Aldrich. Pyranine **P3**, was obtained commercially from Alfa Aesar. Riboflavin 5'-monophosphate **P2**, Methylene blue trihydrate **P6**, sodium 3'-hydroxy-3-oxo-3H-spiro[isobenzofuran-1,9'-xanthen]-6'-olate **P7**, 3,4-Dihydroxy-9,10-dioxo-2-anthracenesulfonic acid sodium salt **P8**, Alizarin-3-methyliminodiacetic acid **P9**, 8-Anilino-1-naphthalenesulfonic acid **P10,** were obtained commercially from Fluorochem. Deionised water (18.2 MΩ cm) was used throughout.

Preparation of 1 L of phosphate buffer (pH 7, 0.02M), the following was as follows: 500 ml of a 0.1 M solution of 5.35 g of Potassium Phosphate Dibasic Heptahydrate (0.0615 M) and 2.61 g of Potassium Phosphate Monobasic Monohydrate (0.0385 M) were prepared adjust solution to pH 7 using HCl or NaOH. A dilution was made for the 0.02 M Buffer pH 7. For one litre of Buffer, 200 ml of the 0.1 M buffer pH 7 was taken and made up to 1 L with water.

Individual UV-Vis spectra were obtained using an Agilent Technologies Cary 100 UV-Vis spectrophotometer. Infrared spectra were obtained using a Shimadzu IRAffinity-1S Fourier Transform Infrared Spectrophotometer. Individual fluorescence spectra were obtained using an Agilent Technologies Cary Eclipse Fluorescence Spectrophotometer. Well-plate fluorescence studies were performed using a BioTek Cytation 5 Cell Imaging Multi Mode Reader. ^{1}H NMR, ^{13}C NMR, HSQC, COSY and DEPT-135 spectra were all obtained using a Bruker Ascend™ 400 MHz NMR spectrometer.

The commercially available graphene oxide samples were all obtained in the UK, from companies in the UK, Europe, and adjacent countries, and analysed without further purification other than protocols described. Most were obtained as aqueous dispersions (1-10 mg/ml); where obtained as solids, these were dispersed in deionised water (18.2 MΩ cm; metals analysis available in ESI Section 4.6).

[Further experimental details are included throughout this document]

2. Synthesis and Characterisation of Probes

2.1. Synthesis of P1 and P5

Probe 1

1-(4-bromobutyl)pyrene

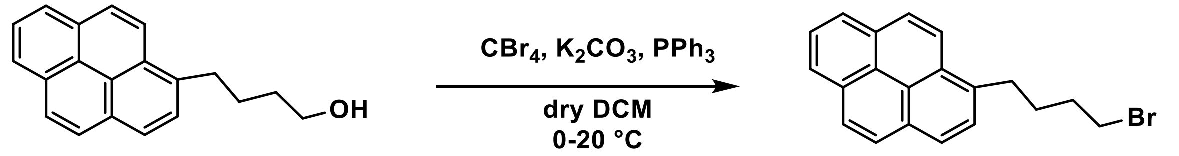

1 **2**

Carbon tetrabromide (1256 mg, 3.787 mmol, 1.3 eq) was added to potassium carbonate (604 mg, 4.37 mmol, 1.5 eq) and then purged with N_2. 1-pyrenebutanol **1** (800 mg, 2.92 mmol, 1 eq) was taken up in dry dichloromethane (15 mL) giving a clear, pale yellow solution. This solution was then added dropwise to the reaction mixture. Triphenylphosphine (920 mg, 3.51 mmol, 1.2 eq) was taken up in dry dichloromethane (5 mL), which was then syringed dropwise into the reaction vessel. The reaction mixture was then cooled to 0 °C and allowed to stir to room temperature for 18 h, giving an amber oil. The crude product was purified by flash chromatography, eluting with a 6:1 hexane : ethyl acetate solvent system to afford **2** [425 mg, 1.275 mmol, 44% yield] as a colourless oil, becoming a pale-yellow solid after being reduced under reduced pressure. ^{1}H NMR (400 MHz; $CDCl_3$) δ 8.26 (d, 1H, *J* = 9.3 Hz, Ar-H), 8.17 (dd, 2H, *J* = 7.6, 3.3 Hz, Ar-H), 8.12 (d, 2H, *J* = 8.5 Hz, Ar-H), 8.06 – 7.97 (m, 3H, Ar-H), 7.86 (d, 1H, *J* = 7.8 Hz, Ar-H), 3.47 (m, 2H, $-CH_2-Br$). 3.36 (m, 2H, $Ar-CH_2$), 2.03 (m, 4H, $Ar-CH_2-(CH_2)_2-CH_2-Br$) ppm; ^{13}C NMR (100 MHz, $CDCl_3$) δ 136.2, 131.6, 131.0, 130.1, 128.8, 127.6, 127.5, 127.4, 126.8, 126.0, 125.2, 125.1, 125.1, 125.0, 124.9, 123.4, 33.8, 32.8, 30.4 ppm.[1]

Sodium (1-pyrenyl)butylsulfonate (P1)

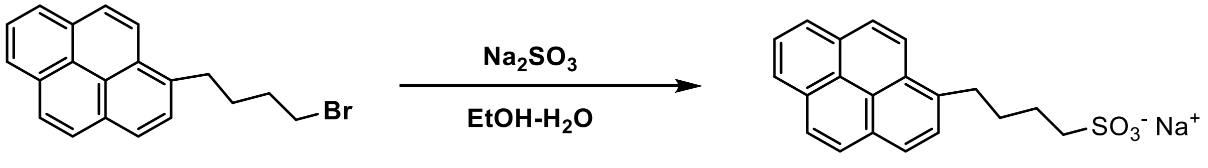

2 **Probe 1**

Sodium sulfite (897 mg, 7.12 mmol, 12 eq) was added to a reaction vessel and purged with N_2. **2** (200 mg, 0.59 mmol, 1 eq) was taken up in portions of ethanol (7.5 mL), which was then sonicated and syringed into the reaction vessel. Water (11 mL) was then added to the reaction vessel, which was heated to 100 °C overnight. The crude reaction mixture was concentrated *in vacuo* and triturated with dichloromethane. The dichloromethane was left to evaporate, leaving a pale-yellow solid, which was then washed with cold water and ethanol, and left to dry under vacuum to yield a pale-yellow solid, **Probe 1** [190 mg, 0.50 mmol, 85% yield]. ^{1}H NMR (400 MHz; DMSO-*d6*) δ 8.37 (d, 1H, J = 9.3, Ar-H), 8.26 (m, 2H, Ar-H), 8.21 (q, 2H, J = 4.1 Hz, Ar-H), 8.12 (m, 2H, Ar-H), 8.05 (t, 1H, *J* = 7.6 Hz, Ar-H), 7.95 (d, 1H, *J* = 7.8 Hz, Ar-H), 3.32 (m, 2H, $-CH_2$-SO3), 2.50 (m, 2H, $Ar-CH_2-$), 1.83 (m, 2H, $Ar-CH_2-CH_2-CH_2-CH_2-SO_3$), 1.74 (m, 2H, $Ar-CH_2-CH_2-CH_2-CH_2-SO_3$) ppm; ^{13}C NMR (100 MHz, DMSO-*d6*) δ 137.1, 130.9, 130.5, 129.2, 128.1, 127.6, 127.5, 127.2, 126.4, 126.1, 124.9, 124.9, 124.8, 124.3, 124.2, 123.6, 51.5, 32.7, 31.0, 25.3 ppm

[1] K. W. J. Heard, C. Bartlam, C. D. Williams, J. Zhang, A. A. Alwattar, M. S. Little, A. V. S. Parry, F. M. Porter, M. A. Vincent, I. H. Hillier, F. R. Siperstein, A. Vijayaraghavan, S. G. Yeates and P. Quayle, *ACS Omega*, 2019, **4**, 1969–1981.

***N,N,N*-trimethyl-4-(pyren-1-yl)-butan-1-aminium bromide**

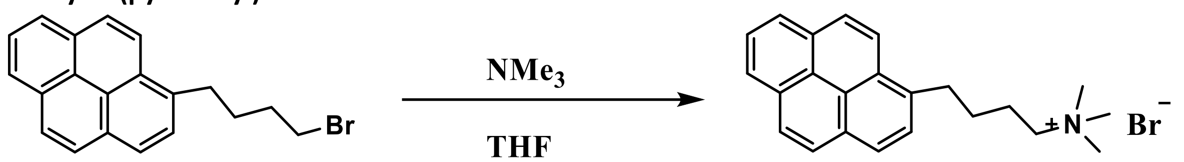

2

2 (150 mg, 0.44 mmol, 1 eq) was dissolved in THF (15 mL) and NMe_3 (33 wt% in EtOH, 5 mL, 21.1 mmol, 48 eq), forming a pale-yellow solution, which was stirred for 2h. Additional NMe_3 (33 wt% in EtOH, 15mL, 63.3 mmol, 144 eq) was added, with the solution being stirred for a further 48 h. An orange mixture formed, from which the product was precipitated using EtOH/H_2O. This was then left to air dry for 96 h and a light brown solid was collected, **Probe 5** [57.9 mg, 0.17 mmol, 37% yield]. ^1H NMR (400 MHz; DMSO-*d6*) δ 8.39 (d, 1H, J = 9.3 Hz, Ar-H), 8.30 – 8.22 (m, 4H, Ar-H), 8.14 (m, 2H, Ar-H), 8.05 (t, 1H, J = 7.6 Hz, Ar-H), 7.99 (d, 1H, J = 7.8 Hz, Ar-H), 3.41 (m, 4H, Ar-H and -CH_2-N(CH_3)$_3$), 3.08 (s, 9H, -CH_2-N-(CH_3)$_3$), 1.89 (m, 2H, Ar-CH_2-CH_2-CH_2-CH_2-SO_3), 1.79 (m, 2H, Ar-CH_2-CH_2-CH_2-CH_2-SO_3) ppm; ^{13}C NMR (100 MHz, DMSO-*d6*) δ 136.6, 131.4, 130.9, 129.9, 128.5, 128.0, 128.0, 128.0, 127.1, 126.7, 125.5, 125.5, 125.3, 124.7, 124.6, 123.4, 65.5, 55.4, 52.7, 32.5, 31.8, 28.7, 22.6 ppm.[2]

[2] M. S. Becherer, B. Schade, C. Böttcher and A. Hirsch, *Chem. Eur. J.*, 2009, **15**, 1637–1648.

2.2 UV-vis and fluorescence spectrum of probes

The UV-vis and fluorescence spectrum was obtaining use a solution of probes at 0.1 mM in phosphate buffer (0.02 M, pH=7.0, filtered 0.2 µm), in a well plate was added to the 33 uL of probes (0.1 mM), 127 uL of phosphate buffer.

UV-vis	Fluorescence
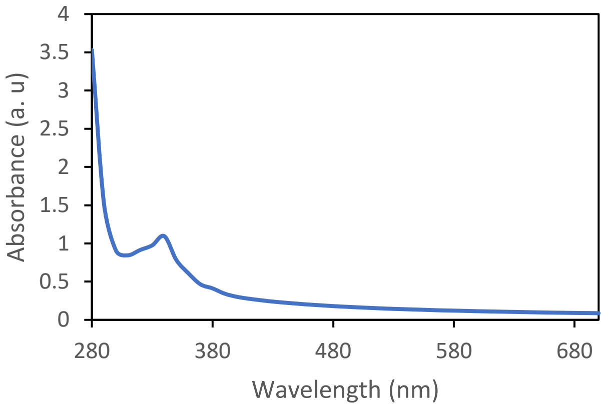	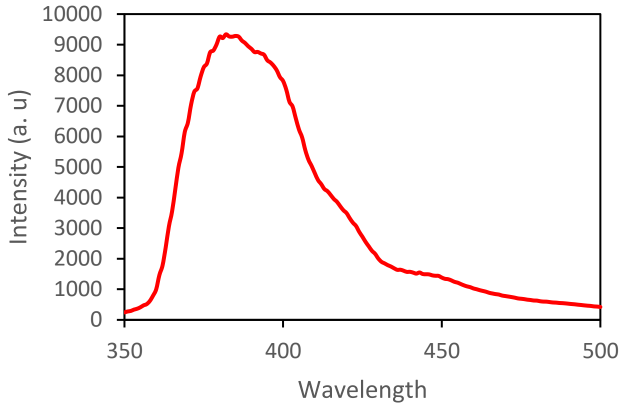
**P1**	
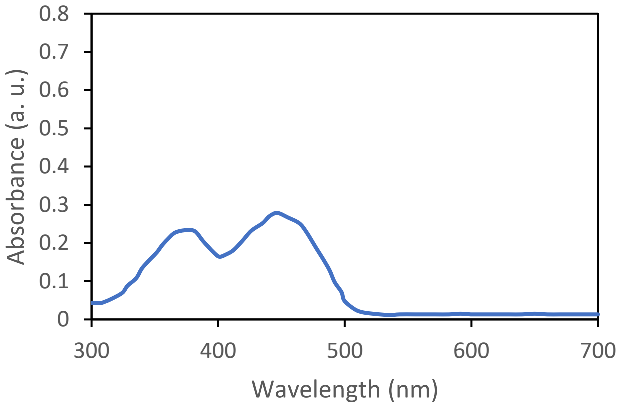	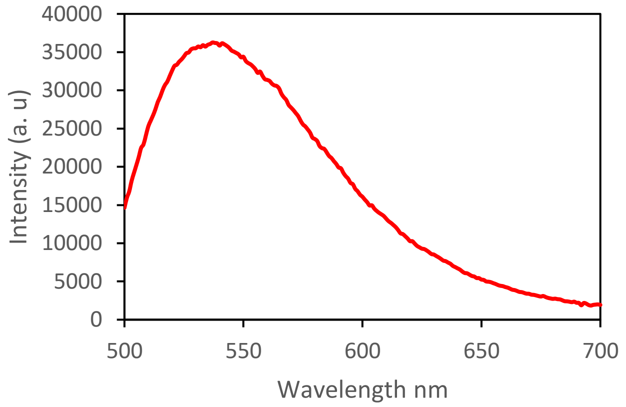
**P2**	
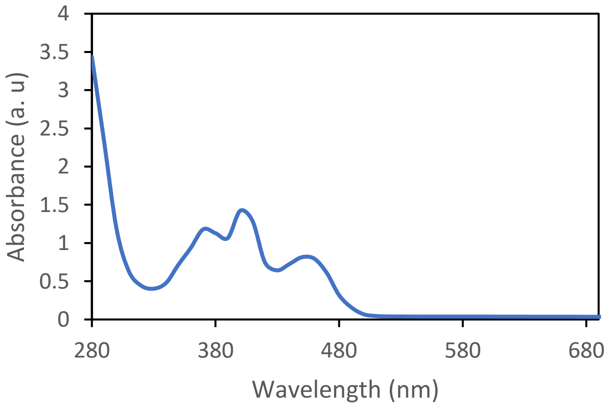	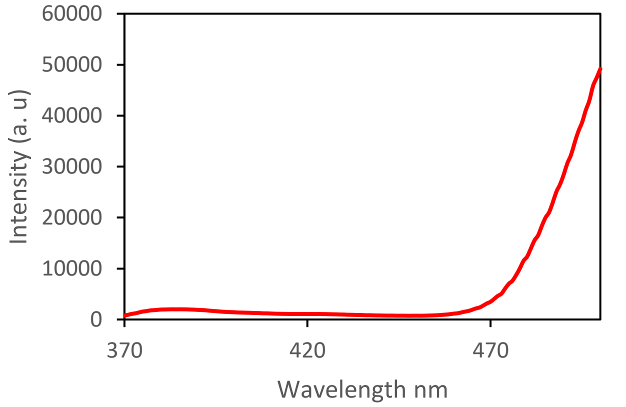
**P3**	

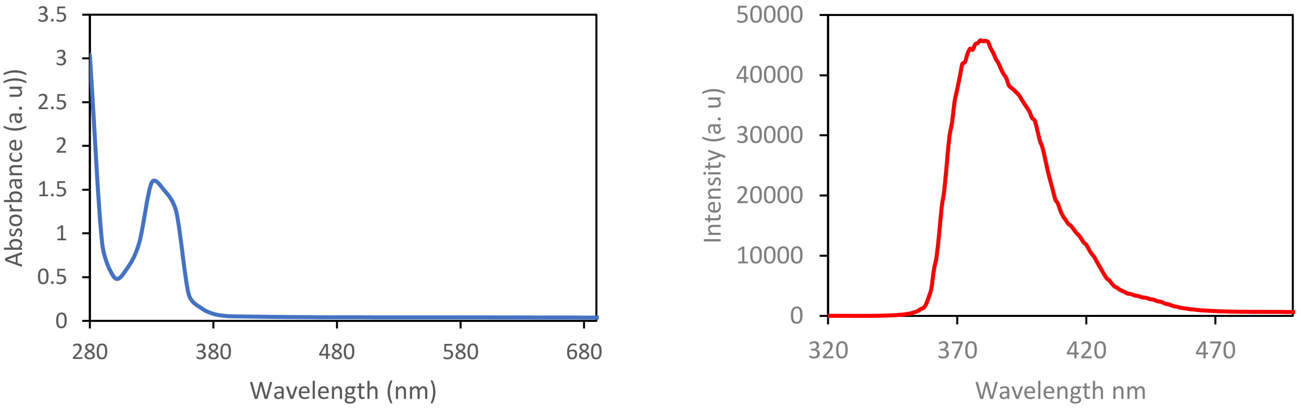
Absorbance (a. u))
Wavelength (nm)
280
380
480
580
680
Intensity (a. u)
Wavelength nm
320
370
420
470

P4

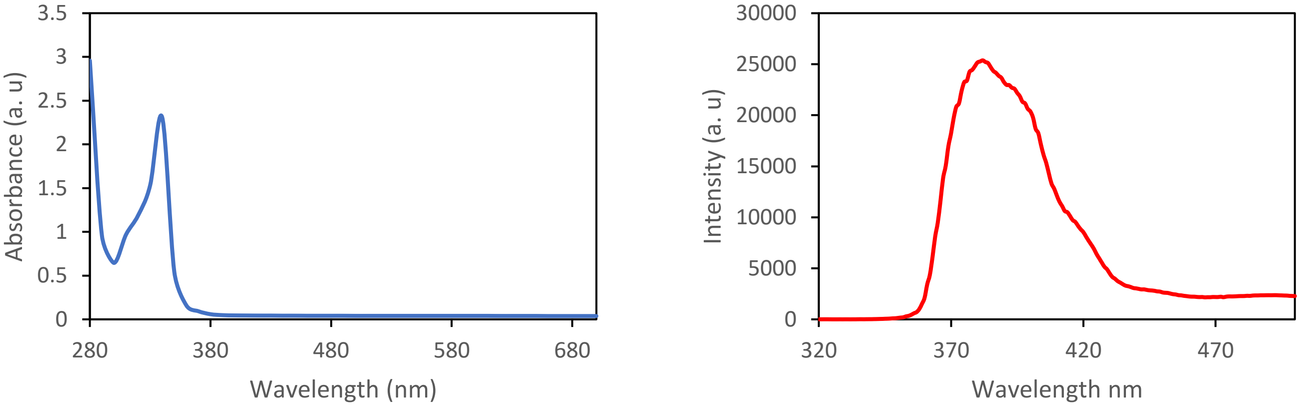
Absorbance (a. u)
Wavelength (nm)
280
380
480
580
680
Intensity (a. u)
Wavelength nm
320
370
420
470

P5

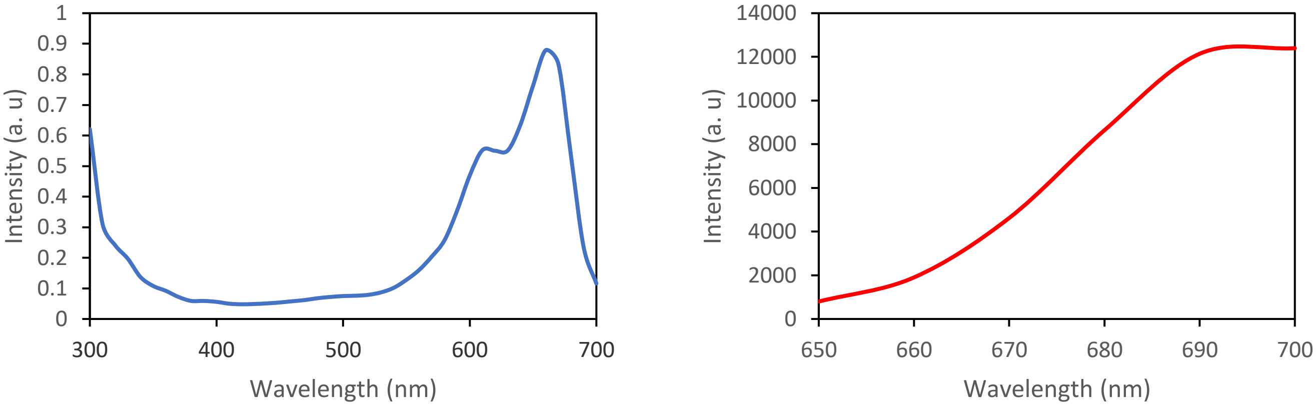
Intensity (a. u)
Wavelength (nm)
300
400
500
600
700
Intensity (a. u)
Wavelength (nm)
650
660
670
680
690
700

P6

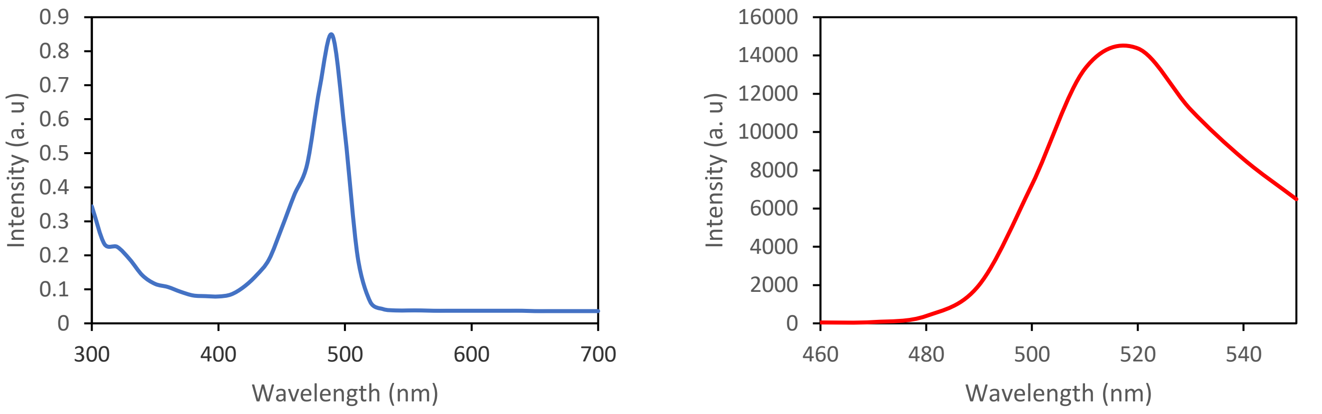
Intensity (a. u)
Wavelength (nm)
0.9
0.8
0.7
0.6
0.5
0.4
0.3
0.2
0.1
0
300
400
500
600
700
Intensity (a. u)
Wavelength (nm)
16000
14000
12000
10000
8000
6000
4000
2000
0
460
480
500
520
540

P7

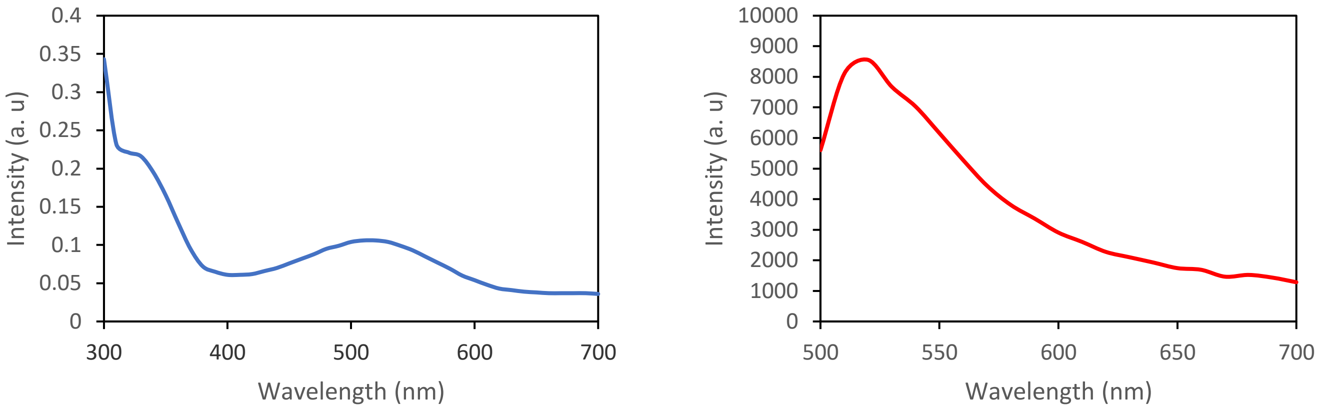
Intensity (a. u)
Wavelength (nm)
0.4
0.35
0.3
0.25
0.2
0.15
0.1
0.05
0
300
400
500
600
700
Intensity (a. u)
Wavelength (nm)
10000
9000
8000
7000
6000
5000
4000
3000
2000
1000
0
500
550
600
650
700

P8

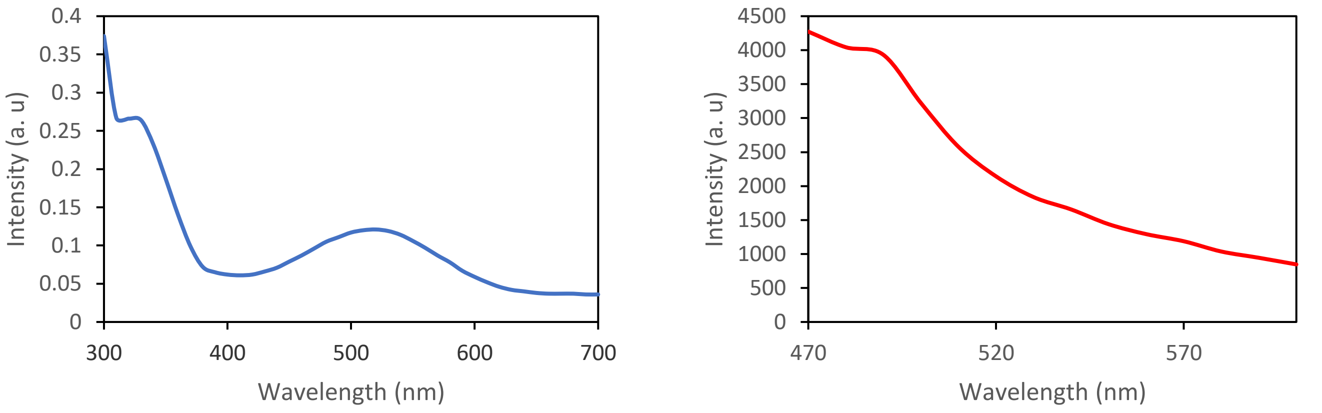
Intensity (a. u)
Wavelength (nm)
0.4
0.35
0.3
0.25
0.2
0.15
0.1
0.05
0
300
400
500
600
700
Intensity (a. u)
Wavelength (nm)
4500
4000
3500
3000
2500
2000
1500
1000
500
0
470
520
570

P9

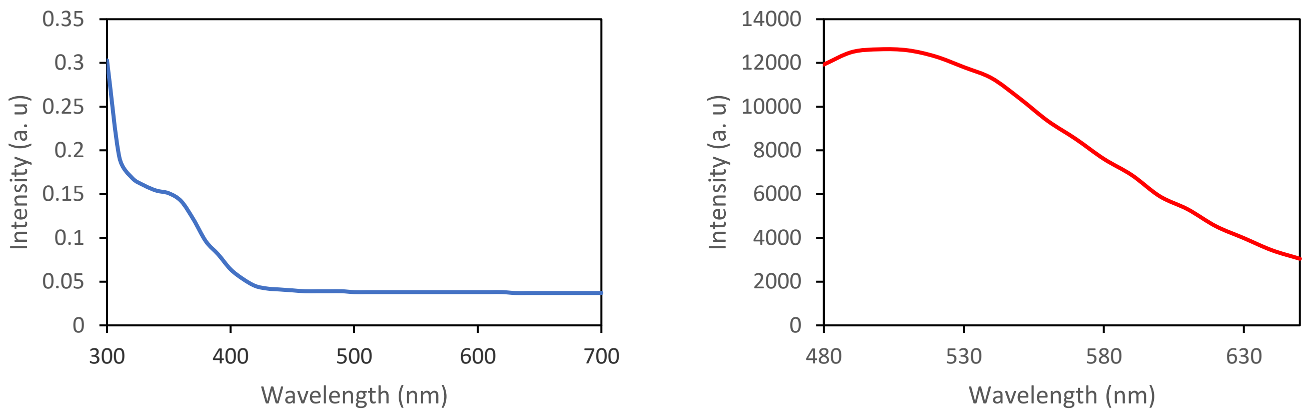

P10

Table S1. The excitation, emission range and emission max wavelength of each probe for fluorescence spectrums.

Probe	λ_{exc} (nm)	λ_{em} (nm)	Em_{max} (nm)
1	320	350-500	390
2	444	500-700	544
3	330	370-500	390
4	270	320-480	390
5	444	500-700	544
6	600	650-700	690
7	321	460-530	520
8	450	500-650	520
9	420	470-600	490
10	360	480-650	500

2.3. Interactional Fingerprinting Assay

Well plate fluorescence studies of GO and data analysis

*Interactional Fingerprinting Assay (*All probes except **P3**)
In a 96-well plate, 127 µL of Phosphate Buffer (0.02 M, pH=7.0, filtered) and 33 µL of each solution probe (0.02M, pH=7.0, filtered) was added and 40 µL of each GO dispersion 0.1 mg/mL in distilled water) was added. The total volume was 200 µL. The GO samples were sonicated for 30 s and left to rest 15 min before addition (and no more than 30 min). This plate was then analysed in a fluorescence plate reader (3 analytical repeats, averaged).

*Interactional Fingerprinting Assay (***P3,** used at a different concentration, following initial optimisation)
87 µL of distilled water and 33 µL of solution **P3** (0.02 M, pH=7.0, filtered) was added to 96 well plate and 80 µL of each graphene oxide dispersion (0.1 mg/mL in distilled water) in a well plate well to total 200 µL. The GO samples were sonicated for 30 s and left rest 15 min before addition (and no more than 30 min). This plate was then analysed in a fluorescence plate reader (3 analytical repeats, averaged).

The intensities of each sample at the wavelength of emission max were collated (Table S1 for excitation and emission wavelengths). Responses are shown on the following page (Table S2). Each assay was typically repeated (experimental repeat) and number of times (see data below), to account for possible error in liquid handling and surface tension effects in microplate wells.

Table S2. Fluorescence response of **P1** to **P10** with all commercially-sourced graphene oxide samples.

GO	P1	P2	P3	P4	P5	P6	P7	P8	P9	P10
GO[A]	10953	70978	1290	238316	4982	1790	452700	1933.33	17	4534
GO[A]	11886	77750	1328	238132	4709	2111	501067	2009.67	16	4789
GO[A]	11639	81638	1236	235158	5321	2120	474485	1921.33	17	4624
GO[A]	11599	84516	1295	227801	4695	2095	490169	1964.67	16	4494
GO[A]	11977	81626	1252	234043	5431	1951	492096	1966.67	18	4603
GO[A]	11501	83621	1356	227550	8589	2118	461984	1909.67	14	4499
GO[A]	12163	86833	1369	236930	5009	1904	479787	1961.33	18	5462
GO[A]	11875	89149	1192	233075	5039	2040	457122	1960.67	17	4764
GO[A]	12351	86389	1253	235117	5009	2175	483837	1969.00	16	4999
GO[B]	14024	47507	1568	246661	7577	5025	493829	1232.33	12	5075
GO[B]	11136	50110	1568	227084	7039	6676	472537	1117.00	15	3739
GO[B]	12908	49960	1611	218754	5962	2530	479560	1089.00	17	3636
GO[B]	12263	58491	1574	236187	7097	7054	506533	1169.33	16	4303
GO[B]	12358	68279	1557	240262	7327	6982	475400	1151.00	15	4197
GO[B]	12027	60518	1535	248610	6106	6721	534287	1254.67	14	4383
GO[B]	12530	65565	1635	230469	6849	6926	483425	1076.33	15	4277
GO[B]	12723	59831	1570	239689	7233	6963	505787	1516.33	16	3898
GO[B]	13621	77147	1698	238968	6839	6860	484955	1193.67	16	4185
GO[C]	2259	3086	296	4709	66	29	42488	699.33	20	1522
GO[C]	2385	3928	361	5778	70	45	40459	675.00	22	1533
GO[C]	2260	5961	361	5571	70	53	39075	661.33	22	1517
GO[C]	2447	3204	260	8716	78	46	39329	728.33	22	1495
GO[C]	2573	3766	249	4956	74	49	48294	723.00	21	1519
GO[C]	2489	3888	302	8210	75	45	46009	699.33	22	1557
GO[C]	2479	7229	366	8709	67	53	39841	684.67	20	1534
GO[C]	2646	6694	484	8830	75	56	58160	692.67	21	1538
GO[C]	2422	6783	302	5851	68	45	54059	759.67	21	1537
GO[D]	2242	235	201	1476	73	3	299	74.00	5	364
GO[D]	2440	230	306	1869	77	2	615	58.33	4	408
GO[D]	2499	204	197	1460	61	9	297	70.00	5	395
GO[D]	2587	244	255	1515	64	8	287	54.33	5	377
GO[D]	2614	222	240	2324	63	13	286	71.00	4	402
GO[D]	2541	198	217	1671	64	4	283	64.00	5	407
GO[D]	2556	216	205	2605	68	4	284	68.67	3	390
GO[D]	2614	226	187	2396	66	13	280	60.33	6	352
GO[D]	2541	183	182	1551	71	2	290	55.67	4	393
GO[E]	15060	93347	1572	283466	10710	4921	555615	2169.67	14	5517
GO[E]	14862	96757	1634	292046	11714	4896	561855	2230.33	17	5810
GO[E]	15012	98757	1579	284419	11384	4099	557786	2160.67	27	6162
GO[E]	15229	98498	1590	287384	12629	5160	579847	2184.67	16	5995
GO[E]	15406	102146	1560	289006	11747	4948	562140	2316.33	17	6273
GO[E]	15592	103141	1582	285944	10307	4809	567056	2121.33	16	6365
GO[E]	15112	100032	1619	295647	11128	4722	595176	2388.67	16	6558
GO[E]	14072	103769	1578	285463	11964	5007	584794	2311.33	17	6519
GO[E]	15032	103432	1706	291869	11332	4970	594957	2353.00	16	6596
GO[J]	13080	92454	1507	260229	8447	3430	555340	2079.67	17	5324
GO[J]	13181	93516	1458	268842	8708	3444	524586	2076.00	16	5687
GO[J]	13257	96700	1382	270030	8468	3245	542234	2055.67	15	5555
GO[J]	13701	98670	1442	267010	8006	3391	537462	2096.67	15	5845
GO[J]	14159	98102	1330	279148	9537	3539	543749	2133.67	20	6045
GO[J]	13535	98374	1389	282716	7879	3521	560724	2240.00	16	5776
GO[J]	14011	100586	1461	255279	8554	4081	535384	2048.67	16	5711
GO[J]	13343	100567	1380	278495	8849	3680	569803	2194.67	18	5403
GO[J]	13701	99174	1478	268854	7308	3271	567270	2201.00	15	6150
GO[k]	12881	91098	1374	244981	8069	3596	516314	1689.00	15	5142
GO[k]	12734	97084	1417	246121	7369	3433	526110	2189.00	19	5407
GO[k]	13136	95991	1386	239186	7940	3757	507955	2014.33	18	4982
GO[k]	12836	98447	1334	255522	7276	3818	515141	2100.00	19	5562
GO[k]	12606	101079	1406	255851	8372	3956	510817	2204.33	18	5378
GO[k]	12614	100561	1351	255120	7827	3571	526040	2076.33	17	5654
GO[k]	13134	98168	1384	252819	7800	3823	526773	2197.00	18	5806
GO[k]	13232	99668	1380	247635	7781	3814	498543	2113.00	18	5534
GO[k]	12804	99587	1386	252865	7540	3686	515350	2109.00	17	5706
GO[L]	15167	106216	1962	330697	17647	16591	647958	2481.67	14	8485
GO[L]	16717	108619	2005	323318	15897	15679	612685	2247.67	14	6828
GO[L]	15946	109148	2037	325369	15248	16087	641661	2329.67	16	7188
GO[L]	15452	108387	2013	322430	18787	15091	654279	2314.00	14	7454
GO[L]	16613	111321	2027	326235	15556	16222	643753	2281.67	16	7310
GO[L]	16748	110932	2008	332221	17983	15641	661070	2333.67	16	7529
GO[L]	16620	111613	2024	322025	15446	15933	625895	2169.67	17	7132
GO[L]	16775	112128	2024	321217	16913	16201	632336	2276.00	17	7392
GO[L]	15555	110053	2005	319975	17021	15845	634398	2425.67	14	7703
GO[M]	15745	104625	2049	323891	15761	15236	662020	2270.67	21	7381
GO[M]	16032	102699	2042	306465	15851	15255	628741	2272.00	17	7232
GO[M]	15549	104376	2001	342018	17165	16227	667802	2425.00	15	8791
GO[M]	15760	105371	2039	311079	16022	15216	645774	2152.33	16	7245
GO[M]	16342	105468	2053	332880	18766	16463	661902	2432.33	15	7937
GO[M]	15792	109073	2023	324214	17349	15724	664175	2380.00	15	10159
GO[M]	16034	104942	2026	319047	15568	15744	635014	2273.33	16	7717
GO[M]	15746	106488	1958	334412	16743	16904	645347	2342.00	20	7851
GO[M]	16760	107742	2032	329896	15974	16650	651451	2464.67	15	8537
GO[N]	16088	107430	1822	314769	13416	11027	633424	2251.67	17	7662
GO[N]	17136	107160	1827	317555	15597	11185	639976	2327.33	15	7590
GO[N]	16325	107577	1842	321949	13817	10683	610557	2251.33	15	7498
GO[N]	15648	108688	1821	317395	12414	10376	637925	2326.67	18	7692
GO[N]	16717	109735	1812	316175	13996	10720	598730	2265.33	18	7725
GO[N]	16262	108094	1871	317078	12885	10884	652987	2470.00	16	7592
GO[N]	17230	112773	1830	300394	13731	10752	622929	2318.00	19	7676
GO[N]	15962	111550	1780	327145	15182	11506	629822	2488.00	17	8495
GO[N]	16033	111619	1836	322707	15105	10443	650246	2332.67	16	8755
P	18298	118175	2141	348720	16591	16892	706825	2465.67	20	9170
P	17665	116181	2195	329962	17712	17102	649222	2279.67	17	8200
P	17045	119229	2153	353442	18738	17758	688445	2410.00	17	9224
P	17652	118013	2193	319799	18755	16531	647255	2269.00	19	8124
P	17953	117770	2171	351480	22047	17681	667207	2545.00	16	8858
P	17391	119504	2164	347154	19929	17040	671151	2422.33	16	10060

3. Multivariate Analysis of Fingerprint Data

The data from interactional fingerprinting assays was processed to perform a dimensionality-reduction following one of three approaches: (A) PCA, (B) LDA, and (C) LDA in which a data from some samples ("training" or "calibration" set) is used to train the LDA model, and then this model is applied to data from a remaining "test set" of samples. In each case, this was performed using a script in Python (see below for more details on each procedure). The from this output was then plotted (adding colour to denote GO sample identity, even when using unsupervised methods), with 95% confidence ellipses plotted for each GO sample.

(A) PCA:

(e.g. Fig 3B in manuscript)

- Fluorescence intensity data for each repeat of each GO/probe combination in an assay was imported from an Excel spreadsheet, and appropriate data frames prepared using the Pandas[3] and Numpy[4] packages.
- Data was scaled using the StandardScaler function from the sklearn.preprocessing package.[5]
- PCA was performed using PCA function from the sklearn.decomposition package.[5]
- The resulting PCA output was plotted using the matplotlib package (axis labels, etc, were modified from .svg output in published versions for clarity) or exported for plotting in OriginLab.[6]

(B) LDA:

(e.g. Fig 3C in manuscript)

- Fluorescence intensity data for each repeat of each GO/probe combination in an assay was imported from an Excel spreadsheet, and appropriate data frames prepared using the Pandas[3] and Numpy[4] packages.
- LDA was performed using LinearDiscriminantAnalysis function from the sklearn.discriminant_analysis package.[5]
- The resulting LDA output was plotted using the matplotlib package (axis labels, etc, were modified from .svg output in published versions for clarity) or exported for plotting in OriginLab.[7]

(C) LDA with a train/test split:

(e.g. Fig 4 in manuscript)

- Data is imported as above, split into two data sets: "training" data, to establish an LDA model, and "test" data, with which to compare this.
- LDA was performed on the training data set, using LinearDiscriminantAnalysis function from the sklearn.discriminant_analysis package.[5]
- The resulting LDA model was then applied to the "test" data, using the model.fit function.
- The resulting output was then plotted using the matplotlib package (axis labels, etc, were modified from .svg output in published versions for clarity).

[3] Pandas development team, pandas-dev/pandas: Pandas, version latest, 2020

[4] C. R. Harris, K. J. Millman, S. J. Van Der Walt, R. Gommers, P. Virtanen, D. Cournapeau, E. Wieser, J. Taylor, S. Berg, N. J. Smith, R. Kern, M. Picus, S. Hoyer, M. H. Van Kerkwijk, M. Brett, A. Haldane, J. F. Del R´ıo, M. Wiebe, P. Peterson, P. G´erard-Marchant, K. Sheppard, T. Reddy, W. Weckesser, H. Abbasi, C. Gohlke and T. E. Oliphant, *Nature*, 2020, **585**, 357–362.

[5] F. Pedregosa, G. Varoquaux, A. Gramfort, V. Michel, B. Thirion, O. Grisel, M. Blondel, P. Prettenhofer, R. Weiss, V. Dubourg, J. Vanderplas, A. Passos and D. Cournapeau, *MACHINE LEARNING IN PYTHON*, 2011, **12**, 2825–2830

[6] Origin(Pro), Version 2022, OriginLab Corporation, Northampton, MA, USA

Apart from the PCA and LDA plots included in the manuscript, we include here the following analyses (referred to in the manuscript).

PCA analysis of GO library 'Interactional Fingerprint' data (2D plot):

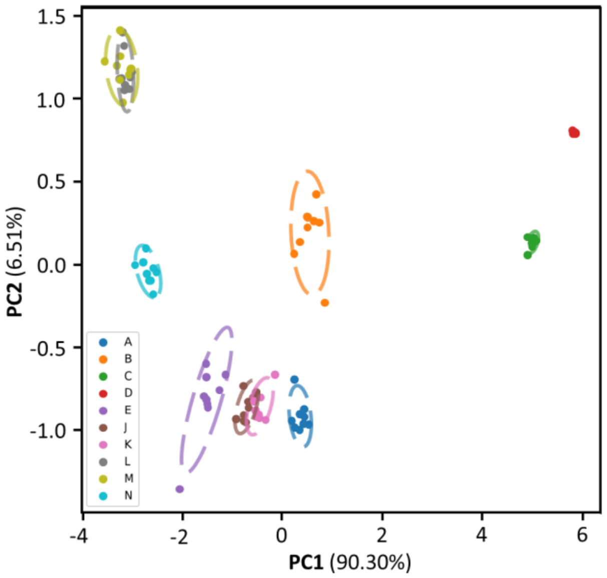

Fig S1. Plot of output from PCA analysis of interaction data from all probes (**P1**-**P10**) with GO library, representing ca. 96.81% of data variance. [Note: PCA is an unsupervised technique. Colours were added for display, and the identity of samples is not included in processing.]

LDA analysis of GO library 'Interactional Fingerprint' data using a reduced probe set (P2, P6, P7, only):

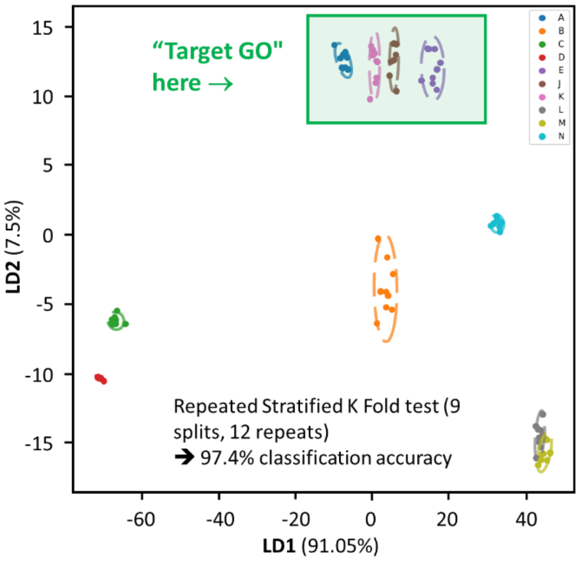

Fig. S2. LDA analysis of interaction data from only three probes (**P2**, **P6**, **P7**) with GO library. Note that this is qualitatively similar to the results from LDA analysis using data from the full probe set, though requiring fewer manipulations (i.e. less time-consuming to perform).

4. Orthodox characterisation on our library of GO materials

The following orthodox characterisations were performed, for comparison with out new approach to characterisation. Where samples were purchased as dispersions in water, they were first lyophilised to remove water, and the resulting solid (stored in desiccator) was analysed without further treatment.

4.1 UV-Vis

Samples were prepared by dilution of 0.001 mg/ml. The UV-Vis absorbance spectra were obtained of all GO samples for triplicate. The wavelength range was from 200 nm to 350 nm. Figure S3 shows the resulting averaged spectrum for each GO sample.

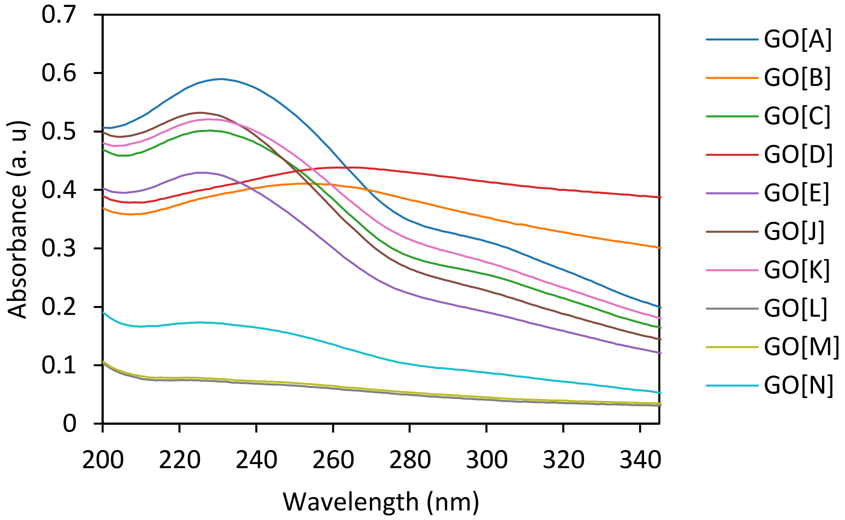

Fig. S3. UV-Vis spectra of GO samples.

4.2 FT-IR

FT-IR spectra were obtained using the ATR accessory for FT-IR. The spectra of all GO dried samples were obtained for triplicate. Figure S4 shows each the averaged spectrum of for each GO sample.

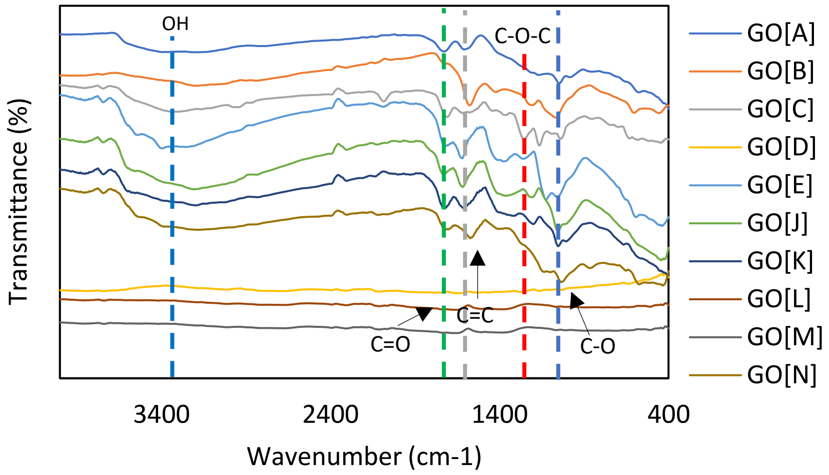

Fig. S4. FT-IR of GO samples.

Bands characteristic of GO were observed in the spectra of **GO[A]**, **GO[B]**, **GO[C]**, **GO[E]**, **GO[J]**, **GO[K]** and **GO[N]**: 1723 cm^{-1}, corresponding to carboxyl groups’ C=O; 1621 cm^{-1}, corresponding to aromatic C=C; 1220 cm^{-1}, corresponding to epoxy groups’ C-O; 1043 cm^{-1}, corresponding to alkoxy C-O; and broad bands at 3391 cm^{-1}, corresponding to hydroxy -OH group (Table S3). In the spectra of **GO[D]**, **GO[L]** and **GO[M]**, none of these signals were observed.

Table S3. Description of each IR spectrum of GO.

GO	Hydroxy –OH (3391 cm^{-1})	Carboxyl C=O (1723 cm^{-1})	Aromatic C=C (1621 cm^{-1})	Epoxy C–O (1220 cm^{-1})	Alkoxy C–O (1043 cm^{-1})
**GO[A]**	X	X	X	Low signal	X
**GO[B]**	X	Low signal	X	X	X
**GO[C]**	X	X	X	X	X
**GO[D]**	-		-	-	-
**GO[E]**	X	X	X	X	X
**GO[J]**	X	X	X	X	X
**GO[K]**	X	X	X	X	X
**GO[L]**	-	-	-	-	-
**GO[M]**	-	-	-	-	-
**GO[N]**	X	X	X	Low signal	X

4.3 XPS

XPS Analysis was performed using a Thermo NEXSA XPS fitted with a monochromated Al kα X-ray source (1486.7 eV), a spherical sector analyser and 3 multichannel resistive plate, 128 channel delay line detectors. All data was recorded at 72W and an X-ray beam size of 400 x 200 µm. Survey scans were recorded at a pass energy of 200 eV, and high-resolution scans recorded at a pass energy of 40 eV. Electronic charge neutralization was achieved using a Dual-beam low-energy electron/ion source (Thermo Scientific FG-03). Ion gun current = 100 µA. Ion gun voltage = 45 V. All sample data was recorded at a pressure below 10-8 Torr and a room temperature of 294 K.

Spectra have been charge-corrected to the main line of the carbon 1s spectrum (adventitious carbon) set to 284.8 eV. All data was recorded at a base pressure of below 9 x 10-9 Torr and a room temperature of 294 K. Data was analysed using CasaXPS v2.3.19PR1.0. Peaks were fit with a Shirley background prior to component analysis. The dried samples were placed on a carbon tape and analysis was carried out in triplicate of each sample.

*Figure S5 shows the XPS survey spectra of **GO[A]** to **GO[N].** In each spectrum the elemental analysis in the sample is shown.*

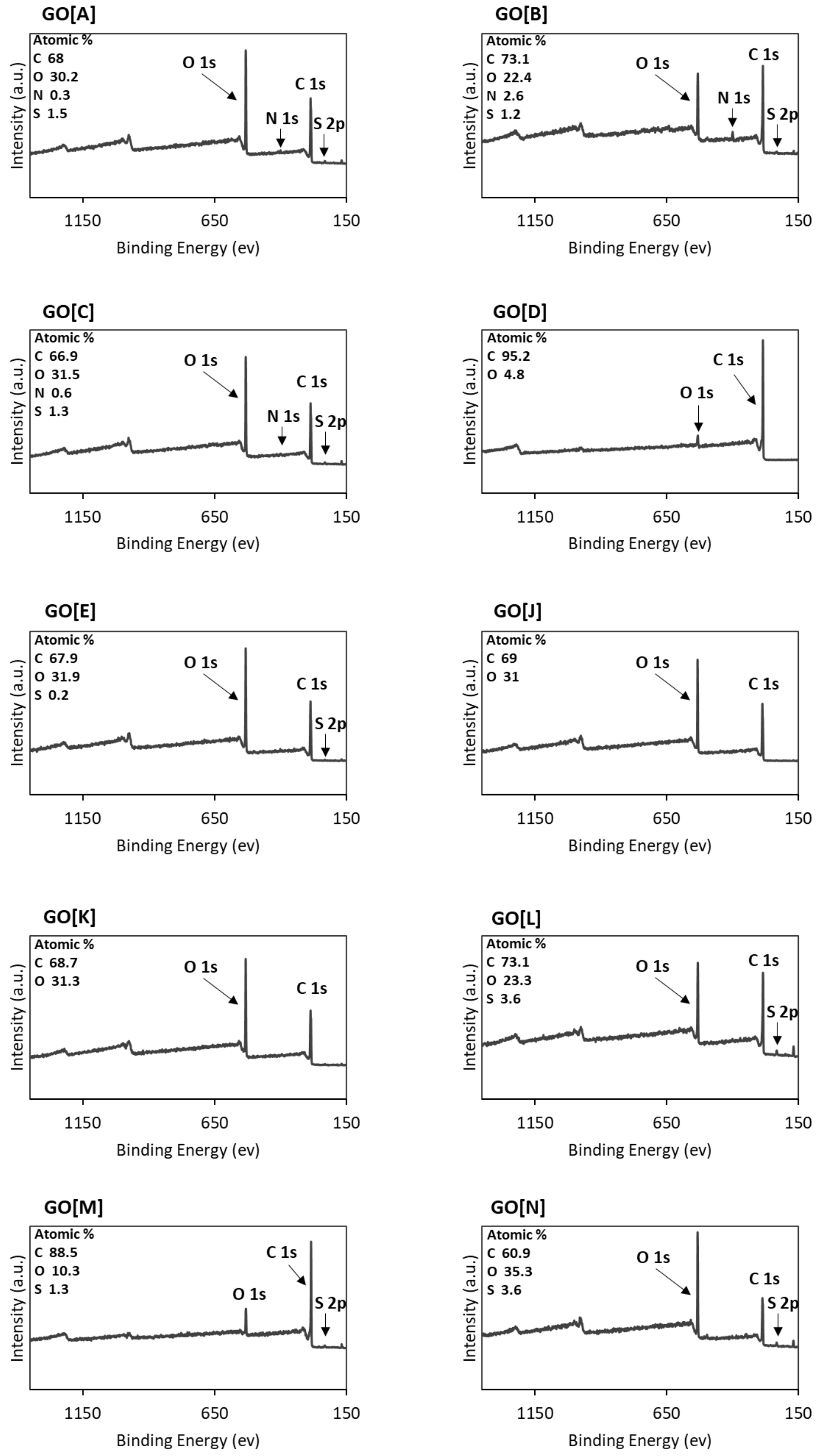

Fig. S5. Survey XPS spectra of graphene oxide samples.

High-resolution C 1s XPS spectra were also recorded for all commercial GO samples. After deconvolution, three distinct peaks, at ~284, ~287, and ~289 eV, can be observed, corresponding to the sp^2 carbon (single C-C and double C=C carbon bonds), the C-, and C=O, respectively (Figure S6). [O/C rate from XPS survey spectra, above].

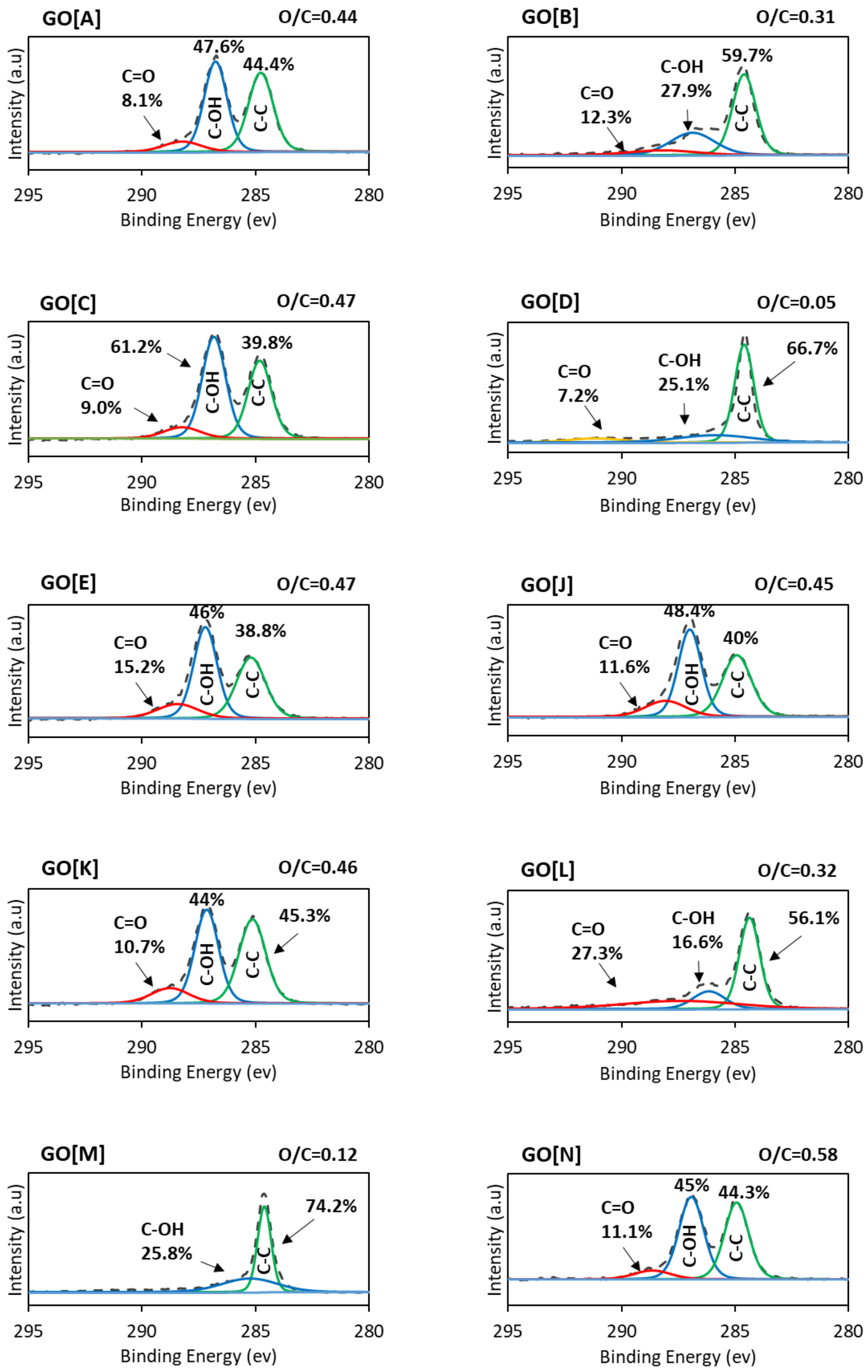

Fig. S6. C 1s XPS spectra of graphene oxide samples.

High-resolution O 1s XPS spectra for GO after deconvolution show 2 peaks: at ~530 eV, corresponding to C=O groups, and ~533 eV, corresponding to C-OH groups (Figure S7).

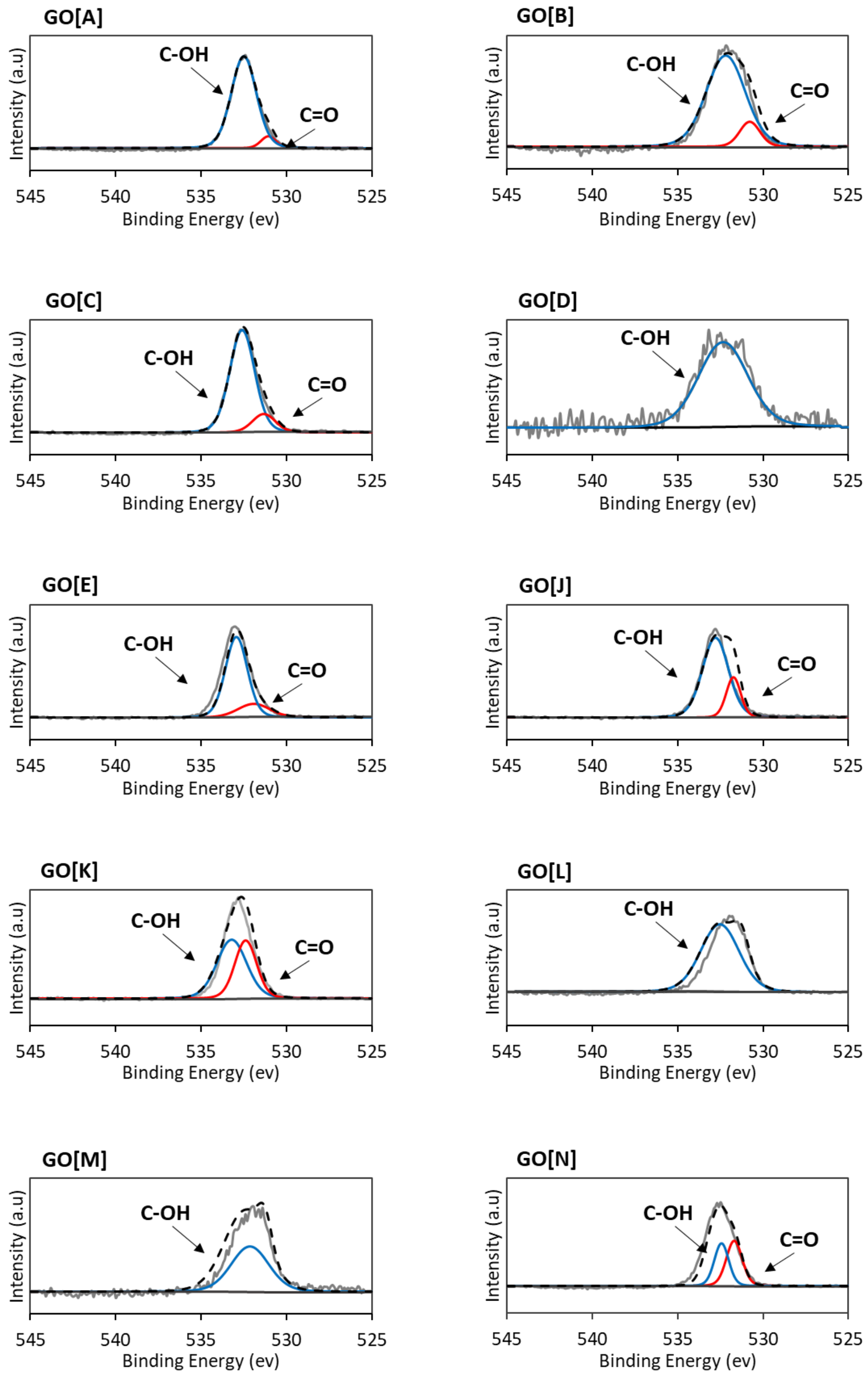

Fig. S7. O 1s XPS spectra of graphene oxide samples.

4.4 Raman spectroscopy

The laser wavelength was 633 nm and the signal was acquired using a 1s integration time of 10 spectra. The measurement was carried out in triplicate of each dried samples. Figure S8 shows Raman spectra of all GO samples. All samples display a D-band at ~1350 cm^{-1}, representative of defects/disorder in the basal plane, and a G-band at ~1590 cm^{-1} representative of the in-plane sp^2 bond stretching. A primary quantifiable Raman measurement is the D/G intensity ratio (I_D/I_G). In most samples, a 2D band around ~2730 is observed.

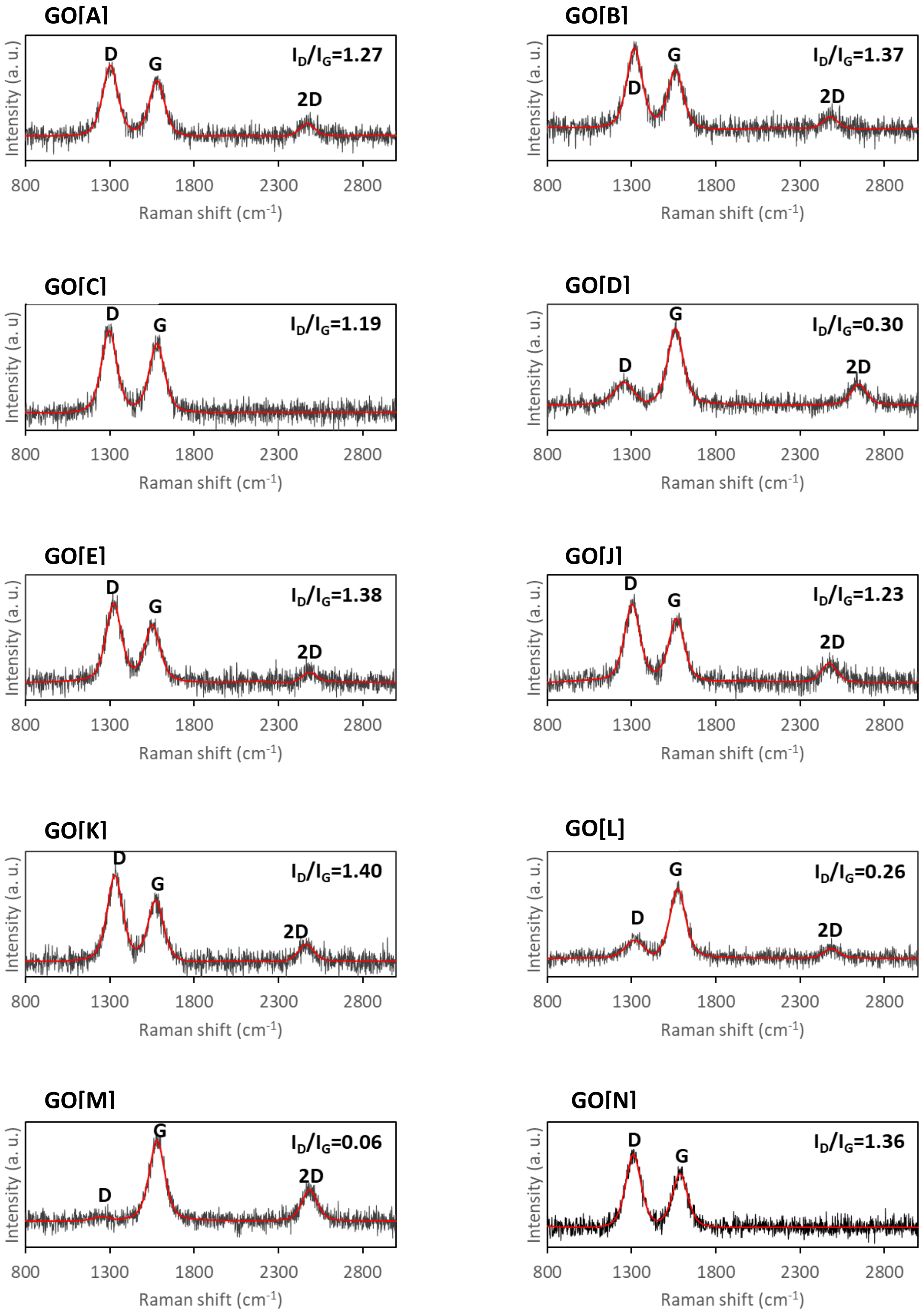

Fig. S8. Raman spectra of graphene oxide samples.

4.5 SEM

A Zeiss Evo SEM was used to characterise the graphene oxide morphology, with an acceleration voltage of 5 kV. Samples were prepared by diluting in water to <0.01 mg/mL to avoid flakes overlapping. Gold-coated glass was mounted on aluminium 25 mm SEM stubs with Cu tape, and single drops of samples were dried for 24 h. Statistical image analysis of SEM data (Table S4) was completed using ImageJ and Origin, where on average 400 flakes from different SEM images were analysed.

Table S4. GO size & morphology by SEM. a) SEM Images of each graphene oxide sample, b) GO flake size distribution fitted with a lognormal distribution (red line) for samples **GO[A]**, **GO[B]**, **GO[D]**, **GO[J]**, **GO[L]**, **GO[M]**, **GO[N]**, and a Gaussian distribution (red line) for samples **GO[C]**, **GO[E]**, **GO[K]**. Samples **GO[C]**, **GO[E]**, **GO[J]**and **GO[K]** and also have a zoom in for smaller GO flakes. c) Histograms of SEM greyscale pixel intensities showing the curves in the 50-200 pixel intensity region. The curves are deconvoluted using a Gaussian distribution to obtain mono- (green), multi- (red)layer distributions; the blue line shows the cumulative curve fit [see Amadei *et. al.*, Carbon, 2018, 133, 398-409].

<table>
<tr><th>Sample</th><th>a) SEM Images of GO Sample</th><th>b) GO Flake Size Distribution</th><th>c) Histogram of SEM Image Greyscale Intensities</th></tr>
<tr><td>GO[A]</td><td>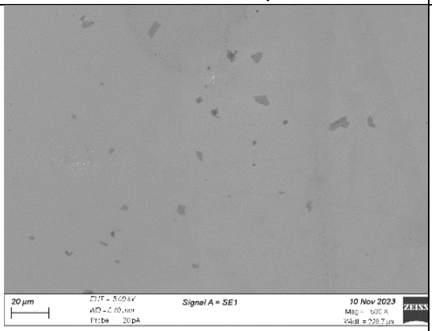
</td><td>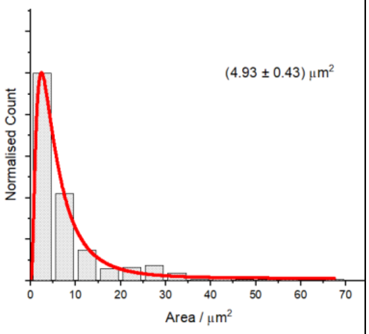
</td><td>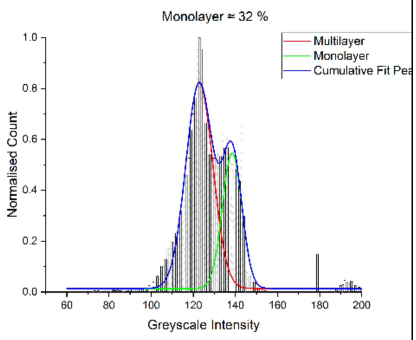
</td></tr>
<tr><td>GO[B]</td><td>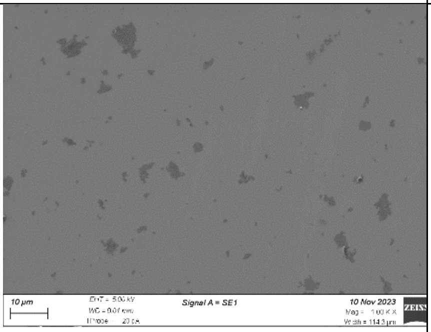
</td><td>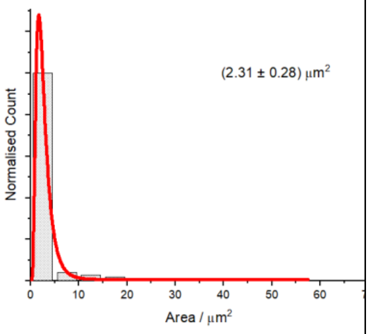
</td><td>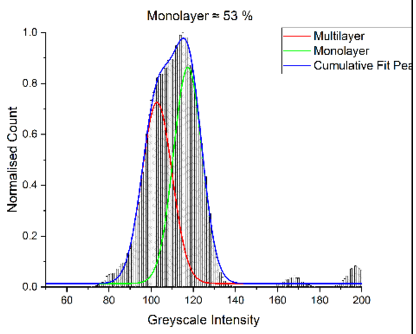
</td></tr>
<tr><td>GO[C]</td><td>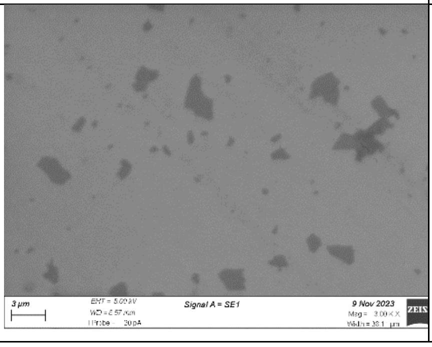
</td><td>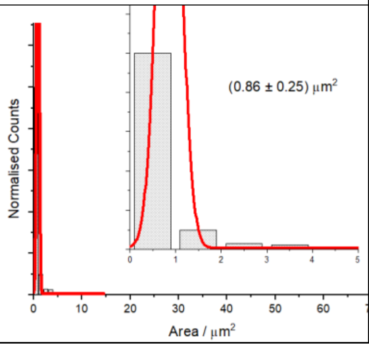
</td><td>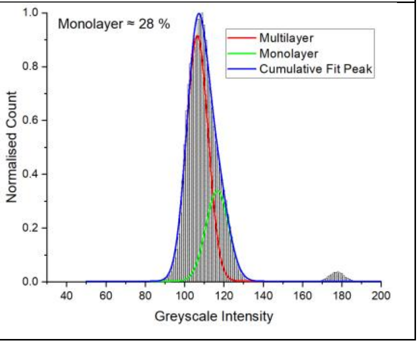
</td></tr>
</table>

**GO[D]**	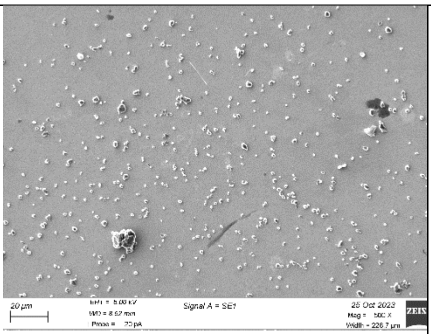	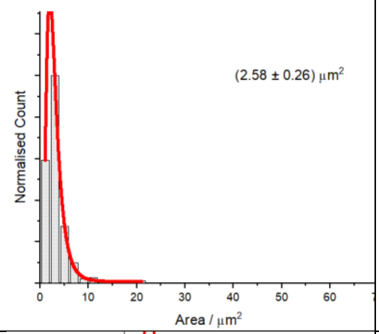	Deconvolution not performed, as flake morphology is not observed
**GO[E]**	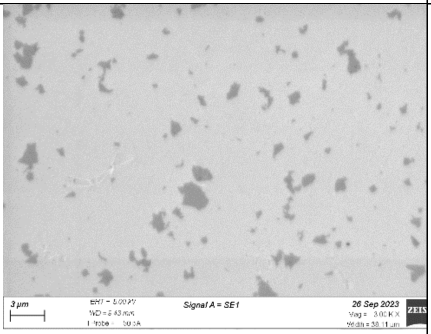	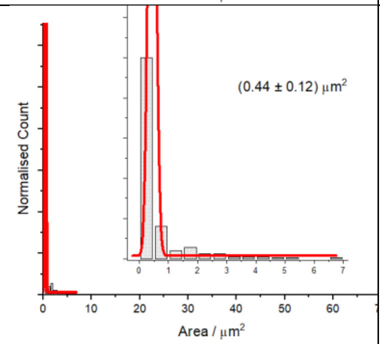	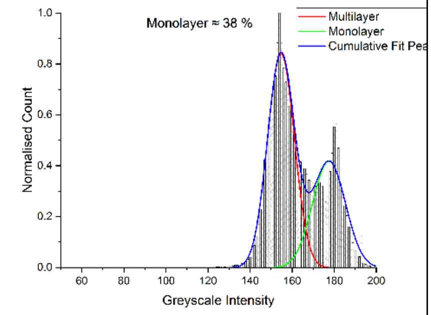
**GO[J]**	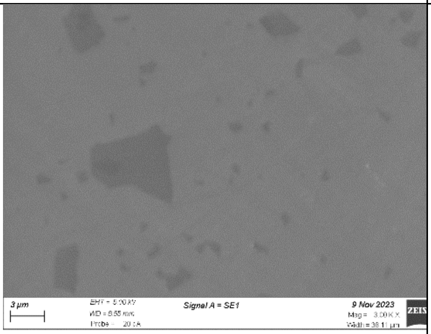	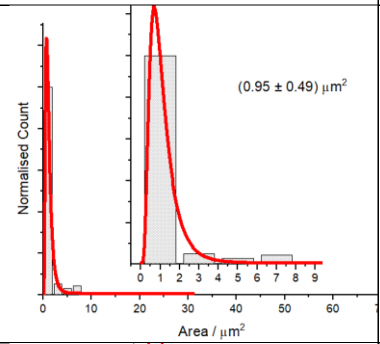	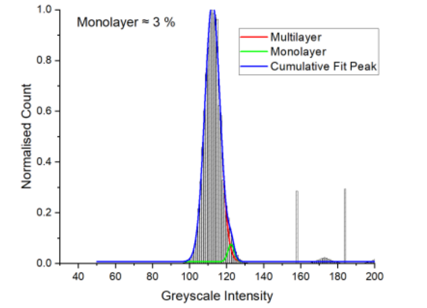
**GO[K]**	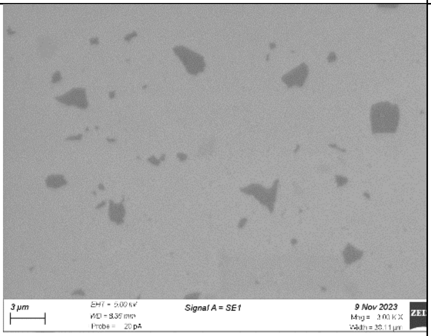	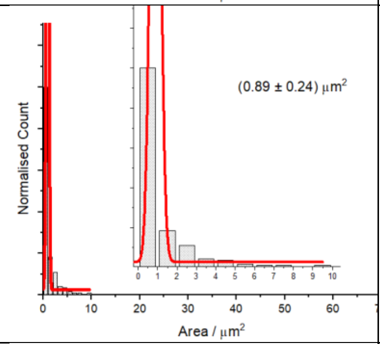	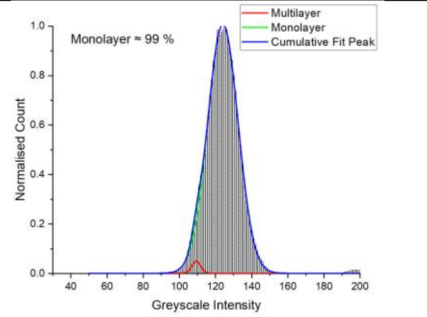
**GO[L]**	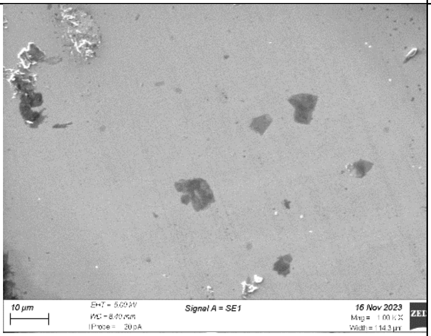	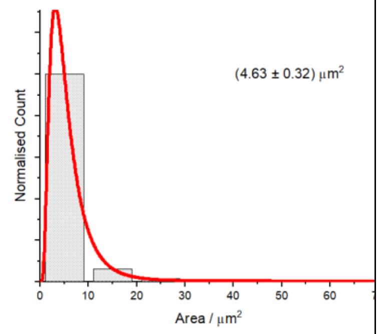	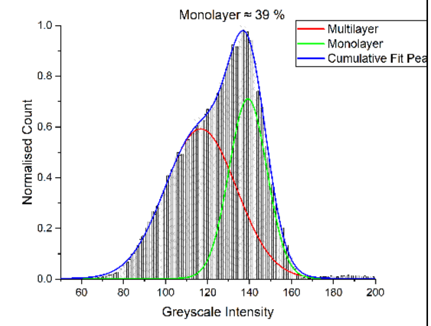

**GO[M]**	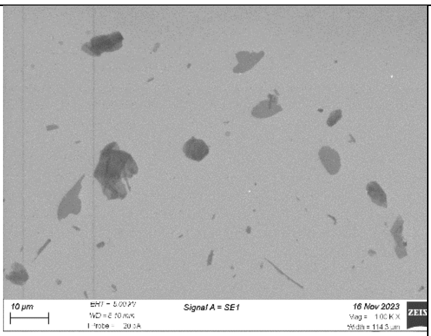10 µm<br>Signal A = SE1<br>16 Nov 2023	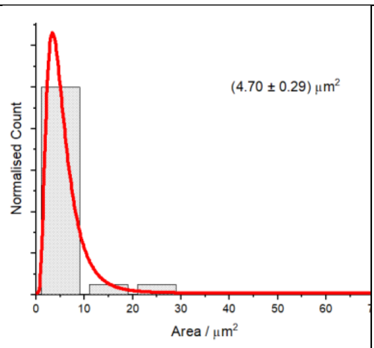(4.70 ± 0.29) µm²<br>Normalised Count<br>Area / µm²	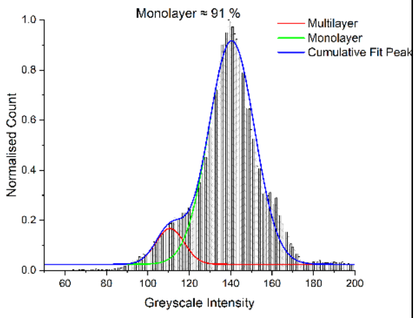Monolayer ≈ 91 %<br>Multilayer<br>Monolayer<br>Cumulative Fit Peak<br>Normalised Count<br>Greyscale Intensity
**GO[N]**	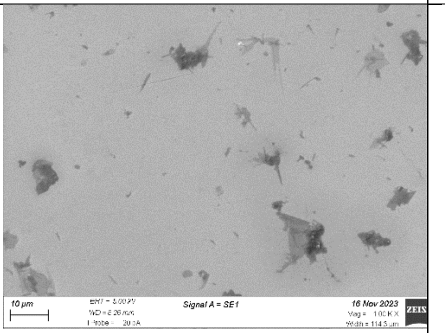10 µm<br>Signal A = SE1<br>16 Nov 2023	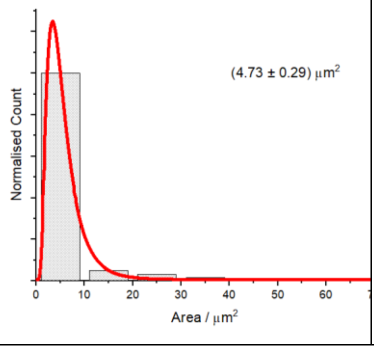(4.73 ± 0.29) µm²<br>Normalised Count<br>Area / µm²	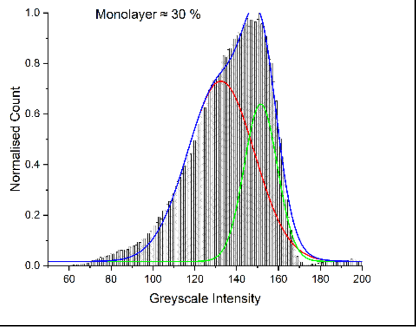Monolayer ≈ 30 %<br>Normalised Count<br>Greyscale Intensity

4.6 ICP-MS

The metallic element compositions of the samples (diluted to 1 mg/ml in Deionised water [18.2 MΩ cm] where supplied as solids or more concentrated suspensions) were determined using inductively coupled plasma mass spectrometry (ICP-MS) in the Imaging and Analysis Centre at the Natural History Museum, London. Samples were prepared by adding 3 mL HNO3 (15.3 M, Romil, SpA) and 1 mL HCl (11.5 M, Romil, Spa) to 1 mL (~1 g) of sample, then digested in a Milestone Ultrawave microwave-assisted digestion system. The resultant solutions were transferred to PTFE containers and evaporated to incipient dryness, before adding 0.56 mL HNO3 (15.3 M, Romil, SpA) and 0.2 mL H2O2 (9.8 M, Merck, Suprapur) and making up to a final volume of 14 mL with Milli-Q water. Sample solutions were analysed on an Agilent 7900 ICP-MS instrument.

	23 Na	24 Mg	27 Al	39 K	44 Ca	51 V	52 Cr	55 Mn	56 Fe	59 Co	63 Cu	66 Zn	75 As	77 Se	137 Ba	140 Ce	195 Pt
	Conc. [ppm]	Conc. [ppm]	Conc. [ppm]	Conc. [ppm]	Conc. [ppm]	Conc. [ppb]	Conc. [ppb]	Conc. [ppb]	Conc. [ppb]	Conc. [ppb]	Conc. [ppb]	Conc. [ppb]	Conc. [ppb]	Conc. [ppb]	Conc. [ppb]	Conc. [ppb]	Conc. [ppb]
**LOQ (ppb)**	8.10	0.851	1.12	7.62	9.65	0.0320	0.176	0.161	1.51	0.0353	0.351	0.926	0.139	0.678	0.097	0.0289	0.0173
**Av. Blank**	0.219	0.178	0.243	0.115	0.0571	1.52	6.33	0.0039	0.218	0.197	0.0164	0.0614	0.229	0.799	4.01	0.208	4.38
**Deionised water**	0.0131	0.0861	0.0545	0.0487	0.0433	<	5.36	0.0162	<	0.0980	0.0245	<	0.0552	<	0.668	0.0041	<
**GO[A]**	0.933	0.572	0.475	0.738	0.490	1.72	46.6	2.72	8.52	8.29	0.0306	0.166	3.09	<	60.2	0.290	0.145
**GO[B]**	1.48	0.210	0.419	0.488	0.0674	1.51	162	0.223	1.22	1.08	0.0200	0.119	0.556	<	4.75	2.39	45.7
**GO[C]**	<	0.132	0.186	1.08	<	1.81	6.82	16.2	0.256	0.358	<	<	0.281	<	1.18	0.0596	<
**GO[D]**	0.341	0.234	0.241	0.197	0.0547	1.53	147	0.0106	1.06	7.37	<	0.0629	0.377	<	5.59	6.21	<
**GO[E]**	0.294	0.186	0.0299	0.812	0.722	<	14.4	0.601	0.152	0.913	0.161	0.147	0.229	<	10.6	<	<
**GO[J]**	2.23	0.118	0.512	0.770	0.113	1.03	117	0.383	1.09	0.703	0.0199	0.190	0.536	<	3.53	0.248	<
**GO[K]**	0.847	0.138	0.491	2.15	0.0560	4.49	6.22	1.49	0.683	0.0776	<	0.009	0.428	<	17.7	0.0448	<
**GO[L]**	5.61	1.26	0.405	7.35	0.688	4.24	294	8.32	11.7	138	0.133	32.9	1.57	1.56	63.5	4.91	16.3
**GO[M]**	3.14	1.00	1.00	3.90	0.545	7.17	232	6.64	13.1	162	0.144	2.10	1.53	0.958	49.4	28.8	28.3
**GO[N]**	6.24	2.07	0.159	2.22	1.01	0.431	4.65	2.02	0.807	3.07	<	0.0413	0.412	0.915	22.4	1.96	<

“LOQ” denotes limit of quantification in the sample

“<” denotes less than LOQ

We note that in assays, probe molecules are present in concentrations >3 mM; alkali metals (phosphate buffer) are present in large excess. By contrast, GO dispersion are diluted to below 4% of the concentration observed here. As a result, the largest concentration of non-alkali metal (Mg) is less than 0.1 mol% that of the probe molecules, and metal concentration may be considered not bind/alter binding of a significant fraction of the probe molecules.

5. Modification of Graphene Oxide surface, by alkylation of surface alcohol groups

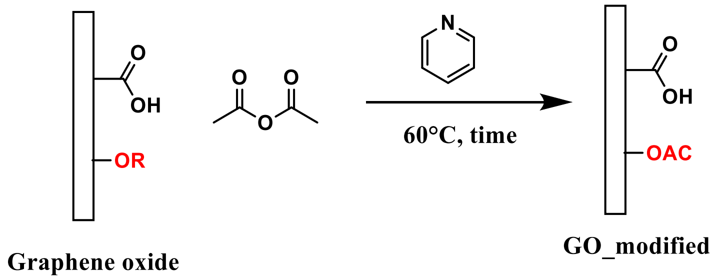

In a 20 ml vial, anhydrous pyridine (5 mL) and different amount of dry acetic anhydride (from 50 µL to 2 mL, depending on the degree of surface modification desired, Table S5) was added to graphene oxide GO[0%)] (50mg), under a nitrogen atmosphere. The reaction was stirred at 60°C for different periods of time (1h to 1 week). Each resulting mixture was filtered, and washed with with diethyl ether, followed by several cycles of washing with warm methanol. The product material was then washed with water, and retained by centrifugation (30 min, 4000 rpm) 4 times. Finally the modified GO was freeze-dried for two days, yielding a fine powder.[7]

Table S5. Conditions of the acetylation reaction. *(*Degree of modification determined by later analysis, see Section 6)*

Identity	Acetic Anhydride	Time	Comments*
**GO[Mod-A]**	0 µL	-	“0% modified”
**GO[Mod-B]**	80 µL	1 week	“19% modified”
**GO[Mod-C]**	165 µL	5 hours	“29% modified”
**GO[Mod-E]**	2 mL	1 hours	“47% modified”
**GO[Mod-D]**	165 µL	24 hours	“Unknown”: sample for which we intend to quantify the degree of surface modification (known to be 38% modified).

[7] A. Talyzin et al., *Phys.Chem.Chem.Phys*, 2020, **22**, 21059-21067

6. Quantification of degree of surface modification using an established quantitative method.

In the established "FLOSS" method,[8] a known amount of a pyrene derivative, which is able to react selectively with alcohol residues on a GO surface, is allowed to react with an analyte sample. The remaining unreacted dye is then washed into a known volume, and its concentration obtained by measuring fluorescence (comparing to a calibration curve). The amount of surface alcohol may be inferred from this: the greater to number of surface alcohol groups, the less unreacted dye is observed following the reaction. We implemented this as follows to determine the proportion (percentage) of GO surface alcohol groups modified by esterification in our series of modified GO samples.

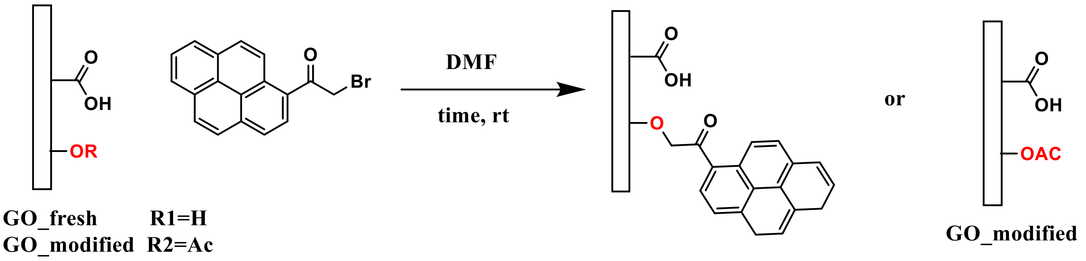

To a 5 mg of sample under study, 5 mL of a 0.412 mM solution of 1-(bromoacetyl)pyrene in DMF and 0.5 mL of triethylamine (TEA) was added. The resulting mixture was stirred in the dark at room temperature for 18 h. The resulting suspensions were centrifuged, and supernatant was transferred to a flask. The remaining sample was washed several times with fresh DMF to remove any dye molecules. The washings were transferred to the same flask and diluted with DMF to 5 mL.

Calibration: Seven samples with different concentrations (0.000137 mM to 0.048 mM) of 1-(bromoacetyl)pyrene in DMF were prepared and the emission spectra were obtained in the fluorometer (excitation wavelength 320 nm, 360 nm to 600 nm) Figure S9.

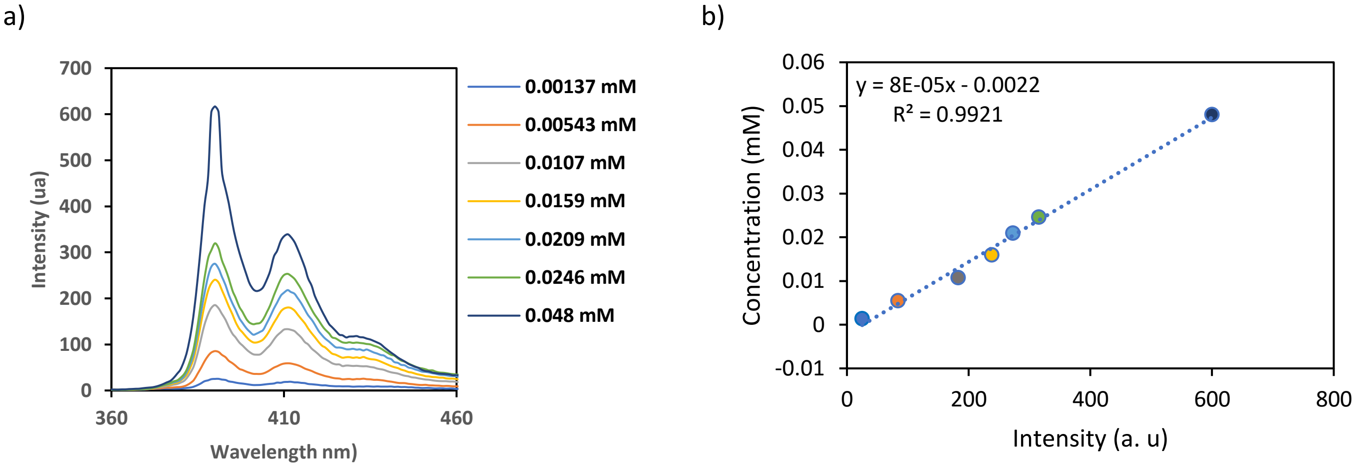

Fig. S9. a) Emission spectra and b) Calibration Curve of 1-(bromoacetyl)pyrene at different concentrations.

[8] Lett. M. Barua et al., *Chem. Phys.*, 2017, **683**, 459–466

The calibration curve obtained is used to calculate the percentage of modification of the OH groups of graphene oxide. The equation used was $y = 8x10^{-5} x - 0.0022$ *(see Fig S9)* where x is the fluorescence intensity at 390 nm and y is the concentration of the dye. The calculations are shown in the following table, and the emission spectra of the reaction washings can be seen in Figure S10.

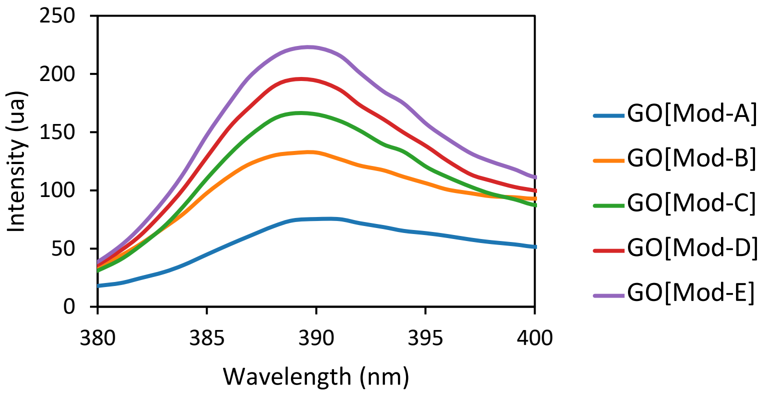

Fig. S10. Emission spectra of graphene oxide modified with acetyl groups.

7. Quantification of degree of surface modification using a modified 'interaction fingerprint' assay.

The following approach was used to demonstrate the potential of our assay to quantify the degree of surface modification (esterification) of the GO samples produced in Section 6 (above):

- Samples with a range of known degrees of surface modification from 0% to 47% (samples **GO[Mod-A]**, **GO[Mod-B]**, **GO[Mod-C]**, **and GO[Mod-E]**) were analysed (as a "training set"), along with a sample whose degree of surface modification we intended to quantify (samples **GO[Mod-D]**, following the methods described earlier (*Section* ***2.3)***.
- PCA was performed (as described in Section **3**).
- Making a plot of *PC1* for each sample (average for all analytical repetitions) against degree of surface modification, shows a linear relationship, suitable for quantification of the degree of surface modification (see Figure S11, below). This may be fit to a straight line (Microsoft Excel).

  e.g. Taking the average PC1 determined for **GO[Mod-D]**, -1.45675, we calculate the degree of modification to be 39.49% using the linear relationship derived from Figure S11 (% Modification = -8.6491($PC1_{average}$) + 26.9). Independently, the % surface modification for **GO[Mod-D]** was determined to be 36%, using the method described in *"2.3. Well plate fluorescence studies of GO and data analysis"*. The discrepancy between the orthodox measurement and this estimation was only 3.9%.

-

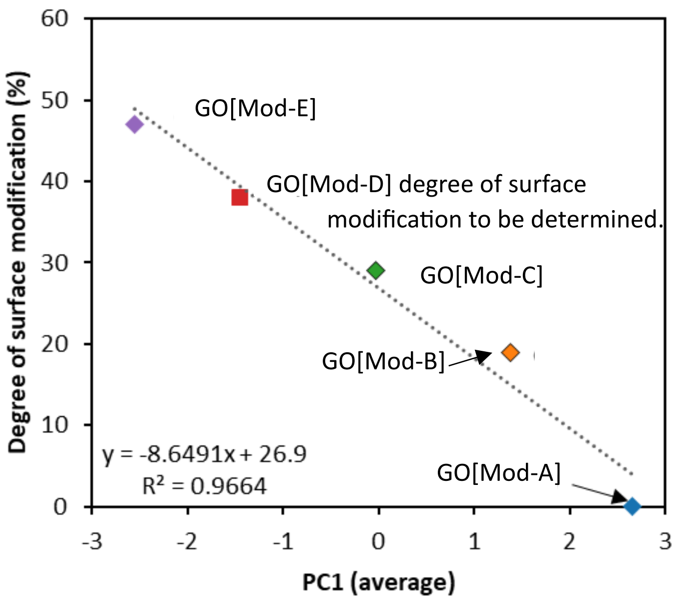

Fig. S11. Calibration curve using PC1(average) and %-GO-Modified.

8. Other orthodox characterisation of graphene oxide modified with acetyl group

8.1 UV-Vis

Samples were prepared by dilution of 0.001 mg/mL (methods described *Section **4.1***). The UV-Vis absorbance spectra were obtained of all GO samples (in triplicate). Figure S12 shows the resulting averaged spectrum for each GO sample.

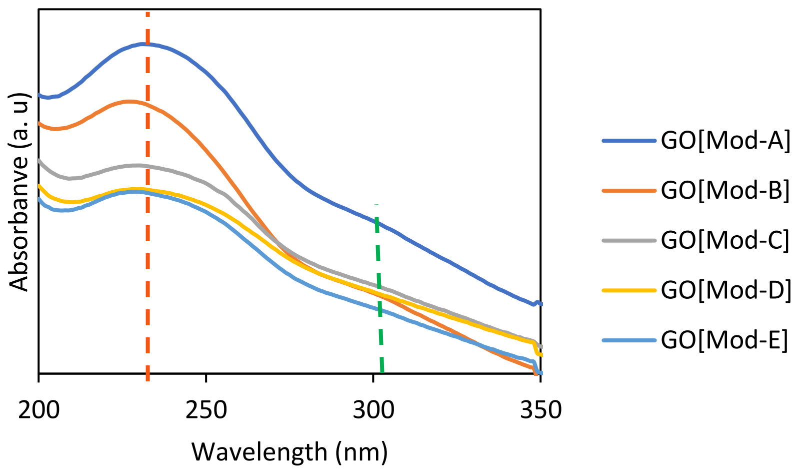

Fig. S12. UV-vis spectrum of GO modified with acetyl groups.

8.2 FT-IR

FT-IR spectra were obtained using the ATR accessory for FT-IR, with spectra recorded in triplicate. Figure S13 shows the averaged spectrum of each GO sample.

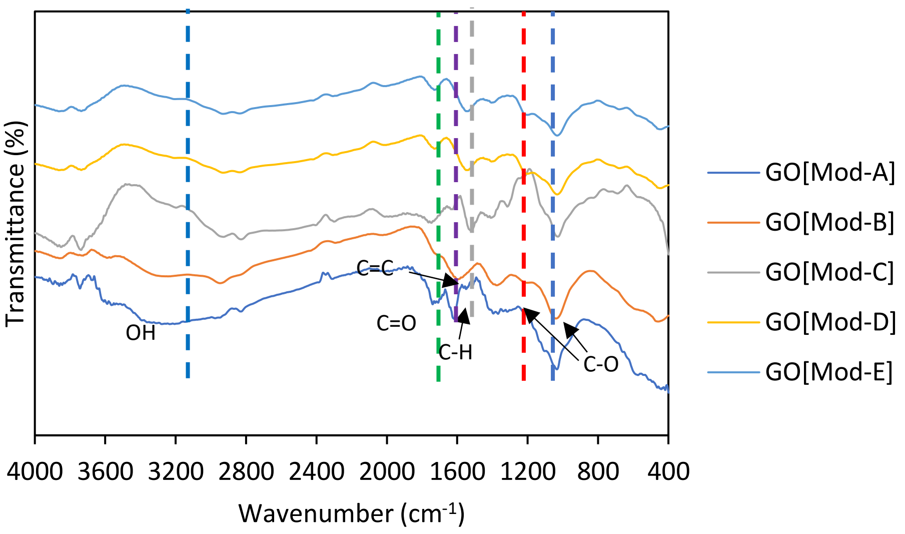

Fig. S13. FT-IR of GO samples.

In the IR spectra of **GO[Mod-A]**, the characteristic bands of GO were observed. At 1723 cm^{-1} common for carboxyl group C=O, 1621 cm^{-1} representatives of aromatic C=C, 1220 cm^{-1}, 1043 cm^{-1} for alkoxy C-O and at 3391 cm^{-1} the broad bands of the -OH group.

The spectrum of GO modified with acetylated groups is different compared to the spectra of **GO[Mod-A]**. However, analysis of the spectra is complicated by the overlap of the main vibrational signatures of acetylation with other peaks of GO that are typically rather broad. For example, To the IR spectrum of **GO[Mod-B]**, **GO[Mod-C]**, **GO[Mod-D]**, and **GO[Mod-E]**, shows the peak of GO at ~1730 cm^{-1}, typically considered as a vibrational signature of C=O of acetyl groups, is also found in precursor GO. Peaks that can be assigned to acetyl group vibrations are also found at 1250 cm^{-1} (C–O) and 1365 cm^{-1} (C–H).

8.3 XPS

The spectra were obtained following the methods described earlier (*Section* ***4.3)***. Figure S14 shows the survey XPS spectra of **GO[Mod-A]** to **GO[Mod-E]**. In each spectra the atomic percentage of elements existing in the sample is shown.

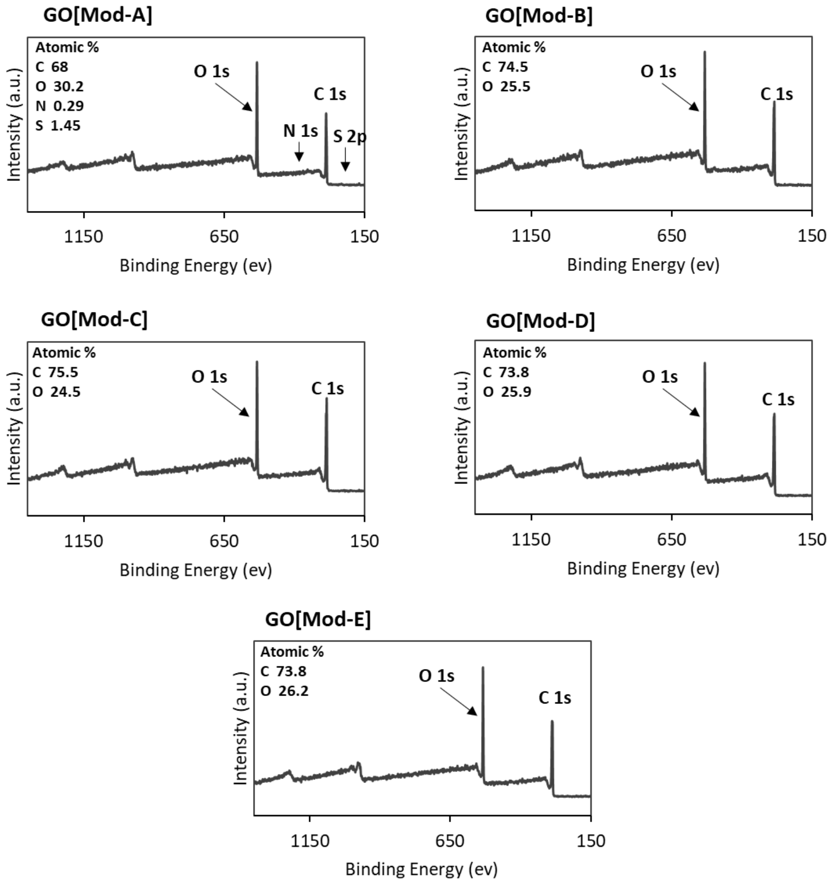

Fig. S14. Survey XPS spectra of graphene oxide modified with acetyl group.

High-resolution C 1s XPS spectra. After deconvolution, three distinct peaks, at ~284, ~287, and ~289 eV, can be observed, corresponding to the sp^2 carbon (single C-C and double C=C carbon bonds), and C-OH, and C=O carbons, respectively (Figure S15). [O/C rate from XPS survey spectra, see above].

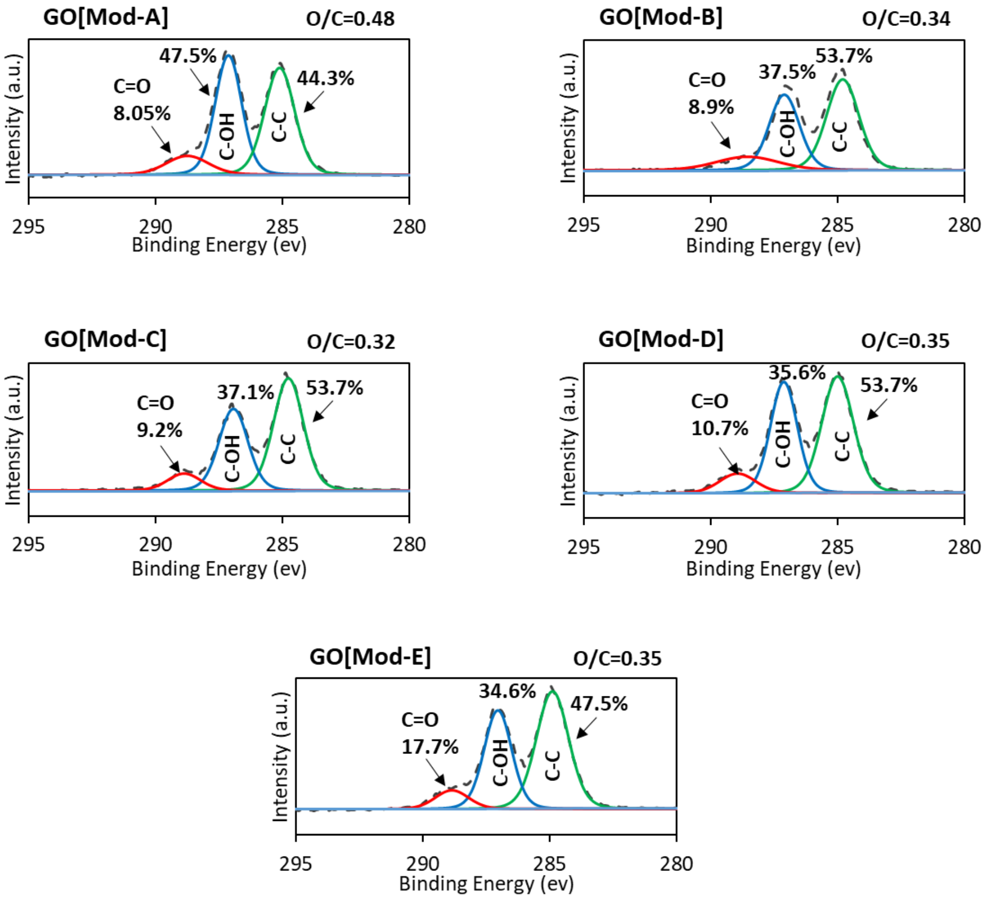

Fig. S15. C 1s XPS spectra of graphene oxide modified with acetyl group.

High-resolution O 1s XPS spectrum for GO after deconvolution shows two peaks at ~530 corresponding to carboxyl group (C=O and OH-C=O) and in ~533 corresponding to alcohol groups (C-OH) (Figure S16).

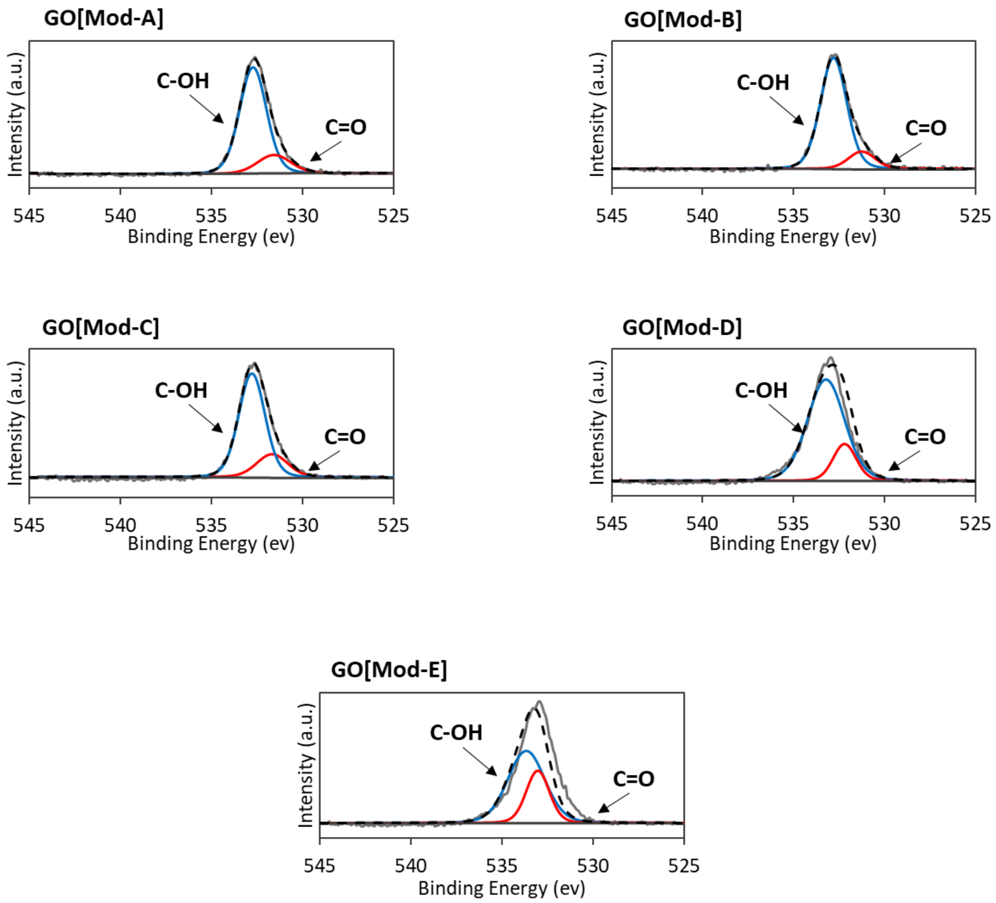

Fig. S16. O 1s XPS spectra of graphene oxide modified with acetyl group.

8.4 Raman spectroscopy

The spectra were obtained following the methods described earlier (*Section **4.4***). Figure S17 shows the Raman spectra to graphene oxide samples. Spectra for all samples include two bands: a D-band at ~1350 cm^{-1}, representative of defects/disorder in the basal plane, and a G-band at ~1590 cm^{-1} representative of the in-plane sp^2 bond stretching. A primary quantifiable Raman measurement is the D/G intensity ratio (ID/IG), which is a measurement of the defect density. In around ~2730 cm^{-1} we observe a 2D peak, common in GO, graphene, and graphite.

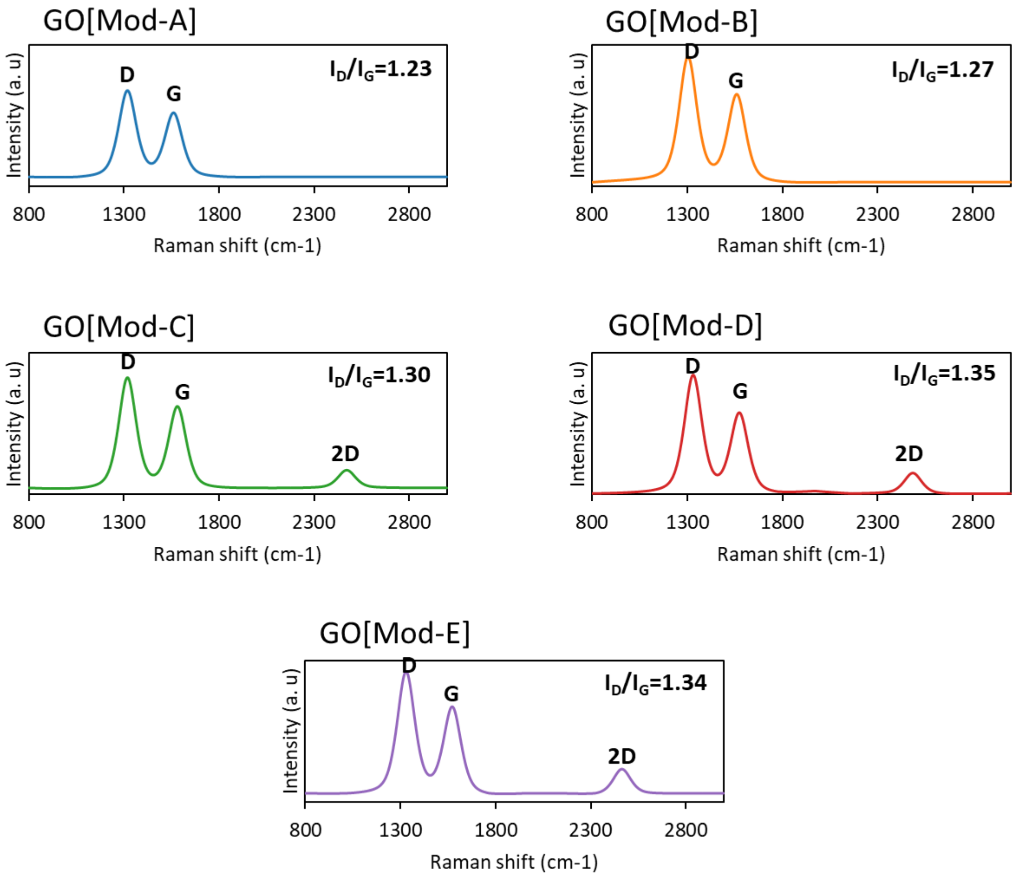

Fig. S17. Raman spectra of graphene oxide modified with acetyl group.
